# Clustered CTCF binding is an evolutionary mechanism to maintain topologically associating domains

**DOI:** 10.1186/s13059-019-1894-x

**Published:** 2020-01-07

**Authors:** Elissavet Kentepozidou, Sarah J. Aitken, Christine Feig, Klara Stefflova, Ximena Ibarra-Soria, Duncan T. Odom, Maša Roller, Paul Flicek

**Affiliations:** 1 European Molecular Biology Laboratory, European Bioinformatics Institute, Wellcome Genome Campus, Cambridge, CB10 1SD UK; 2 Cancer Research UK Cambridge Institute, University of Cambridge, Li Ka Shing Centre, Robinson Way, Cambridge, CB2 0RE UK; 3 Department of Histopathology, Addenbrooke’s Hospital, Cambridge University Hospitals NHS Foundation Trust, Hills Road, Cambridge, CB2 0QQ UK; 4 Division Regulatory Genomics and Cancer Evolution, German Cancer Research Center (DKFZ), Im Neuenheimer Feld 280, 69120 Heidelberg, Germany; 5 Wellcome Sanger Institute, Wellcome Genome Campus, Hinxton, Cambridge, CB10 1SA UK

**Keywords:** CTCF binding evolution, Chromatin architecture, TADs, Cross-species analysis

## Abstract

**Background:**

CTCF binding contributes to the establishment of a higher-order genome structure by demarcating the boundaries of large-scale topologically associating domains (TADs). However, despite the importance and conservation of TADs, the role of CTCF binding in their evolution and stability remains elusive.

**Results:**

We carry out an experimental and computational study that exploits the natural genetic variation across five closely related species to assess how CTCF binding patterns stably fixed by evolution in each species contribute to the establishment and evolutionary dynamics of TAD boundaries. We perform CTCF ChIP-seq in multiple mouse species to create genome-wide binding profiles and associate them with TAD boundaries. Our analyses reveal that CTCF binding is maintained at TAD boundaries by a balance of selective constraints and dynamic evolutionary processes. Regardless of their conservation across species, CTCF binding sites at TAD boundaries are subject to stronger sequence and functional constraints compared to other CTCF sites. TAD boundaries frequently harbor dynamically evolving clusters containing both evolutionarily old and young CTCF sites as a result of the repeated acquisition of new species-specific sites close to conserved ones. The overwhelming majority of clustered CTCF sites colocalize with cohesin and are significantly closer to gene transcription start sites than nonclustered CTCF sites, suggesting that CTCF clusters particularly contribute to cohesin stabilization and transcriptional regulation.

**Conclusions:**

Dynamic conservation of CTCF site clusters is an apparently important feature of CTCF binding evolution that is critical to the functional stability of a higher-order chromatin structure.

## Background

The three-dimensional organization of mammalian genomes comprises distinct structural layers that associate with important functions and range across various scales [1–3]. At a scale of tens to hundreds of kilobases, chromatin is partitioned into topologically associating domains (TADs), which are defined as genomic regions with a high frequency of self-interaction, while few or no interactions are observed between neighboring TADs [4, 5]. As a consequence of their insulating structure, TADs modulate connections between regulatory elements, such as promoters and enhancers, and thus play an essential role in transcriptional regulation [5–9]. TAD structures are reported to be highly conserved across species and cell types [4, 10].

Despite the importance and conservation of TADs, the mechanisms underlying their stability and evolution remain elusive. A large body of evidence supports a model where the CCCTC binding factor (CTCF), colocalized with the cohesin protein complex, plays a causal role in the formation and maintenance of TADs [11–13]. CTCF is a ubiquitously expressed zinc-finger protein with a deeply conserved DNA-binding domain [14–17]. It is responsible for diverse regulatory functions including transcriptional activation and repression as well as promoter and enhancer insulation. Its diverse functions are based on its role in promoting interactions between distant genomic elements by mediating chromatin loop formation [18–20]. A loop extrusion mechanism of TAD formation has been proposed wherein the cohesin protein complex slides along chromatin forming a growing loop until it meets two CTCF molecules bound with convergent orientation. This architecture then prevents cohesin from sliding further, demarcating the TAD boundaries [21, 22]. This model explains why these boundaries usually harbor CTCF binding sites. Nevertheless, there are ubiquitous CTCF-bound regions with diverse functions throughout the genome, while only a small fraction of them occur at TAD boundaries [4]. This has made it challenging to delineate the precise role of CTCF binding in establishing and stabilizing TAD structures.

Several recent perturbational studies experimentally provide some insights into the role of CTCF in determining local and genome-wide three-dimensional chromatin organization. Local disruption of CTCF binding can lead to abrogation of TAD insulation and formation of ectopic *cis*-regulatory interactions between neighboring TADs [5, 8, 13, 20, 23, 24], although TAD structures have been reported to remain intact [5, 21, 25]. Local TAD disruptions may also lead to diseases [26–29]. Upon acute, transient genome-wide depletion of CTCF, there is a marked disruption to chromatin loop and TAD structures [30–32], but the degree of TAD destabilization remains controversial. The impact of this CTCF-mediated insulation on gene expression remains poorly understood. Indeed, experimental approaches that disrupt CTCF binding remain limited by the fundamental roles of CTCF in development and cell viability.

The binding profiles of CTCF in present-day eukaryotic genomes are shaped by repeated waves of transposable element insertions carrying CTCF binding sequences across mammalian genomes [33–36]. Mammalian-conserved sites resulted from ancestral expansions, while recent expansions have established lineage-specific binding patterns. For example, the B2 family of short interspersed nuclear elements (SINEs) active in the mouse-rat ancestor shaped the CTCF binding profile of all Muridae species, and specific members of the B2 family remain active in a lineage-specific manner [33–35]. The human and macaque genomes also share a large fraction of CTCF-associated transposable elements despite the absence of recent large-scale insertional activity [36]. Moreover, representative mammals share conserved CTCF binding sites at their TAD borders [4, 10, 37].

The evolutionary history of CTCF binding facilitates a complementary approach to understanding the role of CTCF in TAD stability. Specifically, we can leverage the natural genetic variation between species as opposed to experimental approaches using targeted or systemic CTCF binding disruption. We can thus investigate the consequences of CTCF binding changes stably fixed by evolution as a version of an in vivo mutagenesis screen [38]. A unique and important advantage of this approach is that the physiological cellular system can be assumed to be in stable and homeostatic equilibrium [39]. CTCF is ideally suited to such an evolutionary approach because in each species the CTCF binding profile is composed of substantial numbers of both deeply conserved and evolutionarily recent sites [34, 35].

Here we performed CTCF ChIP-seq in five mouse strains and species, which have similar genomes and transcriptional profiles, to give insight into the establishment and stability of TADs. Our analysis of the genome-wide CTCF binding exploits natural genetic variation between species to assess the evolutionary dynamics of TAD boundary demarcation. We also investigated how local losses of CTCF binding impact gene expression in the neighboring TADs. We revealed that TAD borders are characterized by clusters of both evolutionarily old and young CTCF binding sites. In addition, CTCF-bound regions at TAD borders, regardless of age, exhibit increased levels of sequence constraint compared with CTCF binding sites not associated with TAD boundaries. Such clusters are consistent with a model of TAD boundaries in a dynamic balance between selective constraints and active evolutionary processes. As a result, they apparently retain a redundancy of CTCF binding sites that give resilience to the three-dimensional genome structure.

## Results

### *Mus*-conserved CTCF binding sites commonly occur at TAD borders

**Fig. 1:**
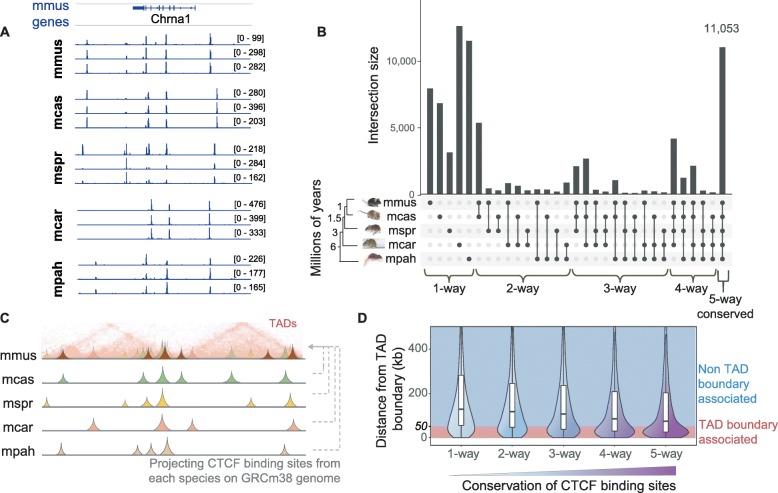
*Mus*-conserved CTCF binding sites commonly occur at TAD borders. **a** CTCF ChIP-seq tracks around the *Chrna1* locus in C57BL/6J and in orthologous regions of the other *Mus* species. The raw data from three independent biological replicates are shown for each species. The majority of peaks are reproducible among the replicates, while a substantial fraction of them is also cross-species conserved. **b** Conservation of CTCF binding sites across the five studied *Mus* species. Conservation levels, i.e., the number of species CTCF sites are shared in, are noted at the bottom of the panel (phylogenetic distances are from Thybert et al. [35] **c** Graphical representation of using orthologous alignments of the CTCF sites identified in each *Mus* species to project them on the genome of C57BL/6J (Mmus, GRCm38) where TADs are available. **d** Distances of CTCF sites with different conservation levels to their closest TAD boundary. CTCF sites with a distance ≤ 50 kb are considered TAD boundary associated, while sites with a distance > 50 kb are referred to as non-TAD boundary associated. For clarity, when referring to the distance to a TAD boundary, we define the boundary as a single nucleotide separating adjacent TADs; when we analyze genomic elements a TAD boundary harbors, we define a window of ± 50 kb around this single nucleotide and refer to this as a “TAD boundary region”

To investigate the evolution of CTCF binding with respect to the boundaries of topologically associating domains (TADs), we experimentally identified CTCF enriched regions in the livers of 5 *Mus* species: *Mus musculus domesticus* (C57BL/6J), *M. musculus castaneus* (CAST), *M. spretus*, *M. caroli*, and *M. pahari* (Fig. 1a, Additional file 1: Figure S1). We characterized the conservation level of the identified CTCF binding sites based on whether they are shared by all species (*Mus*-conserved or 5-way), fewer than 5 species (4-way, 3-way, 2-way), or are species-specific (1-way) (Fig. 1b). The most common categories were the *Mus*-conserved and species-specific CTCF binding sites (Fig. 1b, Additional file 1: Figure S2). We found ~ 11,000 *Mus*-conserved CTCF binding sites, which made up more than a quarter (~ 27%) of the total number of CTCF sites identified in C57BL/6J (Additional file 1: Figure S2). This is consistent with previous observations of high CTCF binding conservation across eutherian mammals, especially compared with other transcription factors such as HNF4A and CEBPA [34, 40, 41]. The vast majority of the *Mus-*conserved sites (92.3%) also had conserved orientations in their CTCF binding motif sequences among all 5 species.

We then intersected the CTCF binding profiles with TAD borders identified from published Hi-C in C57BL/6J liver (Additional file 1: Figure S3) [10]. Although we use Hi-C data for only one of the five species, it has been shown that TADs are largely conserved across species and cell types [4, 11]. For these closely related mouse species with very similar genomes, transcriptomes, and CTCF binding patterns, we expect that this assumption is valid to a great extent. We projected the CTCF sites identified in each of the five *Mus* species onto the C57BL/6J genome assembly (GRCm38/mm10) (Fig. 1c). After grouping all the CTCF sites by conservation level, we measured the distance from each CTCF site to its closest TAD boundary. Based on this distance and the resolution of the TAD map used, we distinguished between TAD boundary-associated (*d* ≤ 50 kb) and non-TAD boundary-associated CTCF binding sites (*d* > 50 kb). We observed that, although CTCF sites of all conservation levels associate with TAD boundaries, more highly conserved CTCF sites were, on average, located closer to TAD boundaries (Fig. 1d). Overall, 41% of the *Mus*-conserved CTCF sites, as compared to 23% of species-specific sites, were found to lie within 50 kb of TAD boundaries (Additional file 1: Figure S4). Our finding of a progressive evolutionary trend between TAD boundaries and CTCF binding conservation, even among closely related species, supports previous reports that shared human-mouse [37] and mouse-dog binding sites overlap with the boundaries of TADs [10].

Shifting the perspective from CTCF-bound regions to TAD boundaries, we found that the majority of TAD borders overlap with highly conserved CTCF binding sites. Nevertheless, a small fraction of the boundaries did not harbor any *Mus*-conserved CTCF binding events. In particular, 12% had CTCF sites conserved only in one, two, or three out of the five studied *Mus* species (Additional file 1: Figure S5). Furthermore, nearly 5% of TAD boundaries apparently do not overlap with any CTCF occupancy (Additional file 1: Figure S5). One potential interpretation is that, although the connection between CTCF binding and TAD boundaries was consistently observed, it may not be a strictly necessary feature for demarcation of TAD boundaries [3].

In summary, the majority of CTCF binding sites are conserved across five mouse species. Moreover, 41% of *Mus*-conserved CTCF binding sites were associated with a TAD boundary, while the vast majority (> 95%) of all TAD boundaries have at least one CTCF binding site.

### CTCF binding sites at TAD boundaries are under strong evolutionary constraint

**Fig. 2:**
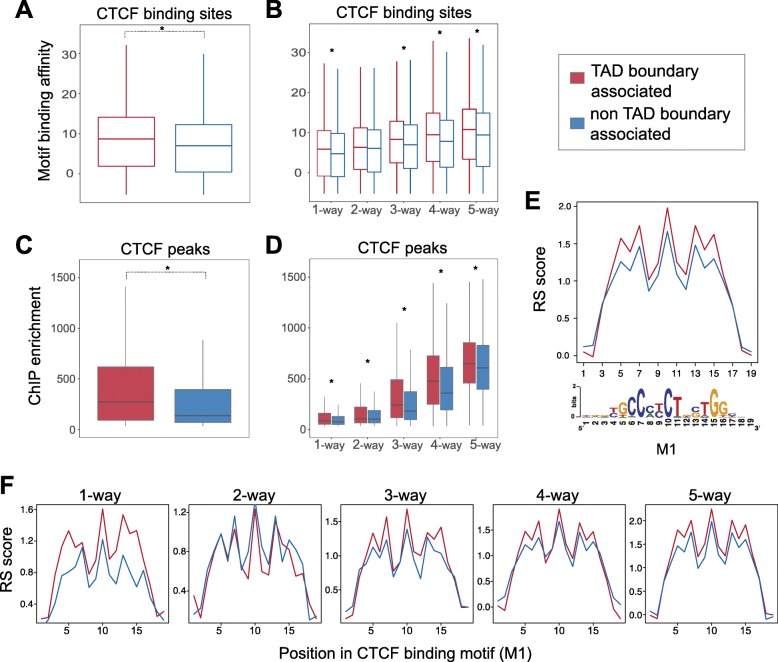
CTCF binding sites at TAD boundaries are subjected to stronger evolutionary constraints. **a** CTCF-bound sites at TAD boundaries contain motifs with a higher binding affinity for CTCF than non-TAD boundary-associated sites (Mann-Whitney *U* test: *p* value < 2.2e−10). **b** Although the binding affinity of CTCF sites is generally proportional to the conservation level of the site (how many species it is shared by), CTCF sites at TAD boundaries have stronger binding affinity than non-TAD boundary-associated sites, independent of their conservation level (Mann-Whitney *U* tests between TAD boundary-associated and non-TAD boundary-associated sites: *p*_1-way_ = 0.001, *p*_2-way_ = 0.06, *p*_3-way_ = 6.1e−07, *p*_4-way_ = 5.2e−13, *p*_5-way_ = 3.9e−11). **c** TAD boundary-associated CTCF peaks display higher ChIP enrichment scores, as calculated by MACS, than non-TAD boundary-associated peaks (Mann-Whitney *U* test: *p* value < 2.2e−10). **d** TAD boundary-associated CTCF peaks, at every conservation level, display stronger ChIP enrichment than non-TAD boundary-associated peaks (Mann-Whitney *U* tests: *p*_1-way_ < 2.2e−16, *p*_2-way_ = 0.002316, *p*_3-way_ < 2.2e−16, *p*_4-way_ < 2.2e−16, *p*_5-way_ = 2.047e−12). **e** The most information-rich bases of the primary CTCF M1 motif at TAD boundaries display higher rejected substitution (RS) scores compared to non-TAD boundary-associated motifs. The bottom panel shows the position weight matrix of the CTCF M1 motif from Schmidt et al. [34] **f** The observation in **e** is independent of the conservation level of the CTCF sites, as shown for subsets of sites at each conservation level

To investigate the role of the TAD boundary association in shaping the characteristics of CTCF binding sites, we first assessed the relationship among CTCF conservation level, TAD boundary association, and CTCF motif strength. Specifically, we identified CTCF motifs from our ChIP-seq peaks and calculated their binding affinity (see the “Methods” section). CTCF is known to bind to a 33/34-bp region of the genome consisting of a primary sequence motif (M1) and a shorter secondary motif (M2) [34]. We found that overall binding affinity, as computationally predicted from the motif sequence, was significantly greater for boundary-associated CTCF sites compared to non-boundary-associated sites (Mann-Whitney *U* test, *p* < 2.2e−16) (Fig. 2a). We asked whether this increase in affinity is driven by the fact that many *Mus-*conserved CTCF sites overlap with TAD boundaries. Although the predicted motif binding affinity increased with the CTCF binding site conservation level, TAD boundary-associated CTCF binding sites consistently had a greater binding affinity than non-boundary-associated sites (Mann-Whitney *U* tests between TAD boundary-associated and non-TAD boundary-associated sites: *p*_5-way_ = 3.9e−11, *p*_4-way_ = 5.2e−13, *p*_3-way_ = 6.1e−07, *p*_2-way_ = 0.06, *p*_1-way_ = 0.001) (Fig. 2b). In addition, we confirmed that, independent of conservation level, CTCF binding sites at TAD borders show higher ChIP enrichment (Fig. 2c, d) and higher counts of mapped reads (Additional file 1: Figure S6) than non-TAD boundary-associated CTCF sites, consistent with the stronger predicted affinity for CTCF. Overall, our results give new insight into the observation that mammalian-conserved CTCF sites have higher motif affinity than species-specific sites [10, 34]. Importantly, for all CTCF binding sites, including species-specific ones, proximity to a TAD boundary was associated with an increase in binding affinity (Fig. 2b, d). This implies that CTCF binding motifs at TAD boundaries may be under a stronger selective constraint than the motif sequences of non-TAD boundary-associated CTCF peaks.

To investigate this hypothesis, we explored evolutionary sequence constraint of the CTCF binding motif itself. We estimated sequence constraint by measuring the rejected substitution rate (RS score) at each position of every 19 base-long primary CTCF binding motif (M1) and compared the score between (a) TAD boundary-associated and (b) non-TAD boundary-associated regions (Fig. 2e, f). RS score is a measure of sequence constraint and reflects the number of base substitutions that were rejected at a specific genomic position as a result of purifying selection, compared to the number of substitutions that would have occurred if the sequence was evolving under neutral selection [42]. We found that the M1 motif in TAD boundary-associated sites displayed higher RS scores compared to the motifs of non-TAD boundary-associated sites (Fig. 2e). We further compared the mean RS score per base between the two categories for CTCF sites at every conservation level and confirmed the generality of this observation (Fig. 2f). We also established that this observation was not caused by an enrichment of specific motif instances at TAD boundaries (Additional file 1: Figure S7).

Taken together, CTCF binding sites at TAD boundaries are subject to stronger evolutionary constraints than the CTCF binding sites that are located further away and this relationship is independent of evolutionary origin of the site.

### LINEs and LINE-derived CTCF sites are under-represented at TAD boundaries

**Fig. 3:**
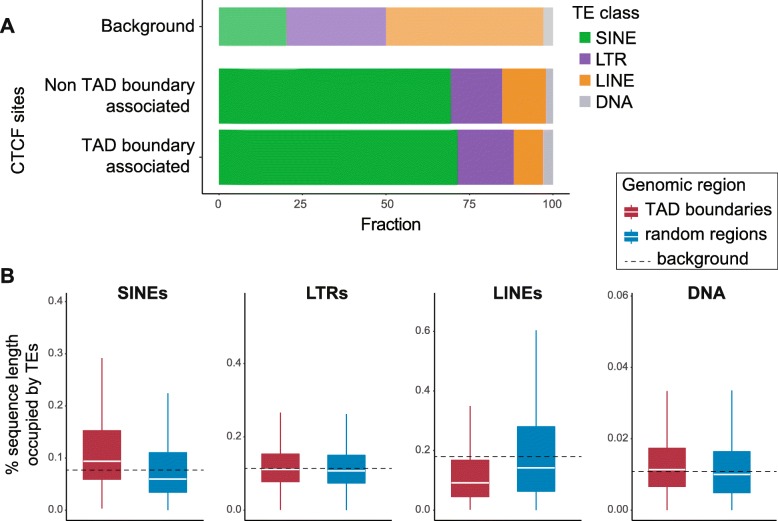
Representation of TE classes and their association with CTCF binding sites differs between TAD boundaries and other genomic regions. **a** Fractions of TAD boundary-associated versus non-TAD boundary-associated CTCF binding sites that are embedded in different TE classes. LINE-embedded CTCF sites are under-represented at TAD boundaries (*χ*^2^ test without Yates correction: *p* = 3.12e−15), while DNA transposon-embedded CTCF sites are over-represented (*χ*^2^ test: *p* = 0.0003), although accounting for just 3% of the TAD boundary-associated sites. SINE-derived CTCF sites (*χ*^2^ test: *p* = 0.01) and LTR-associated CTCF sites (*χ*^2^ test: *p* = 0.015) show no significant differences between the two categories. The top bar shows the percentage of the C57BL/6J genome sequence that corresponds to each TE class, for reference. **b** Fraction of sequence length of TAD boundary regions (TAD boundary ± 50 kb) occupied by each TE class, compared to random genomic regions of equal length. SINE sequences are significantly over-represented (Mann-Whitney *U* test: *p* < 2.2e−16), while LINEs are significantly depleted at TAD boundaries (*p* < 2.2e−16). DNA transposons are slightly, but significantly, enriched at TAD borders (*p =* 9.72e−14), although they account for only 1% of the sequences of the studied regions on average. Representation of LTR sequences shows no significant difference between TAD boundaries and random genomic regions (*p =* 0.005; significance threshold, 0.001)

Having observed that localization of CTCF sites at TAD boundaries affects their sequence and functional conservation, we questioned whether CTCF binding near TAD boundaries appears to evolve by specific mechanisms. Previous results demonstrate that the binding profile of CTCF in eukaryotic genomes is, to a large extent, the consequence of repeat element expansion [33–35, 43]. We searched for potential differences in the transposon classes that drive CTCF binding expansion at TAD boundaries compared to the whole genome. We grouped the CTCF sites based on whether they locate at TAD boundaries or not, and for each group, we calculated the number of CTCF peak centers that were embedded in SINEs, long terminal repeats (LTRs), long interspersed nuclear elements (LINEs), and DNA transposons. As expected, the greatest fraction of CTCF sites in both categories was found to be SINE-derived (Fig. 3a) [33]. The fraction of SINE-derived CTCF sites at TAD borders was slightly, but not significantly, larger than in the rest of the genome (*χ*^2^ test without Yates correction: *p* = 0.01), implying that SINEs may have uniform potential to establish a CTCF site at both TAD boundaries and other genomic regions. Similarly, CTCF sites of LTR origin did not show significant differences between the two categories (*χ*^2^: *p* = 0.015). In contrast, the relative proportion of DNA transposon-derived CTCF sites was increased at TAD boundaries (*χ*^2^: *p* = 0.0003) but accounted for less than 3% of the TEs that contribute to CTCF binding (Fig. 3a). The depletion of LINE-derived CTCF binding sites at TAD boundaries compared to the background genome was the most striking difference (*χ*^2^: *p* = 3.147e−15; Fig. 3a) suggesting that CTCF binding site formation via LINE expansion is significantly less common at TAD borders than genome-wide.

We further assessed the representation of SINE, LTR, LINE, and DNA transposon sequences around TAD boundaries, independent of whether they carry CTCF binding sites. In particular, we determined the fraction of the 100-kb TAD border regions occupied by different transposon classes and compared these with random genomic regions of similar size and distribution. SINE sequences were significantly enriched at TAD boundaries (Mann-Whitney *U* test: *p* < 2.2e−16; Fig. 3b) [4]. The fraction of LTR-derived sequences at TAD boundaries was only marginally higher than random genomic regions (*p* = 0.005), and the fraction of DNA transposon sequences was also slightly higher at TAD borders (*p* = 9.72e−14; Fig. 3b). In contrast, LINE sequences were significantly under-represented at TAD boundaries, compared to random genomic regions (Mann-Whitney *U* test: *p* < 2.2e−16; Fig. 3b), suggesting that TAD boundaries are depleted of LINEs, which may explain why LINE-derived CTCF sites appear under-represented at TAD boundaries (Fig. 3a). Considering the characteristic length of LINE elements, this observation potentially indicates that the insertion of long sequences such as LINEs is negatively selected at TAD borders. This result is complementary to recent reports of selection against long sequence deletions at the functional regions of TAD boundaries [44]. Moreover, it extends our previous observations and reinforces the hypothesis that in addition to TAD boundary-associated CTCF sites being subjected to stronger sequence and functional constrains, TAD boundary regions as a whole are under stronger evolutionary pressure [44].

### TAD borders harbor clusters of conserved and non-conserved CTCF binding sites

**Fig. 4:**
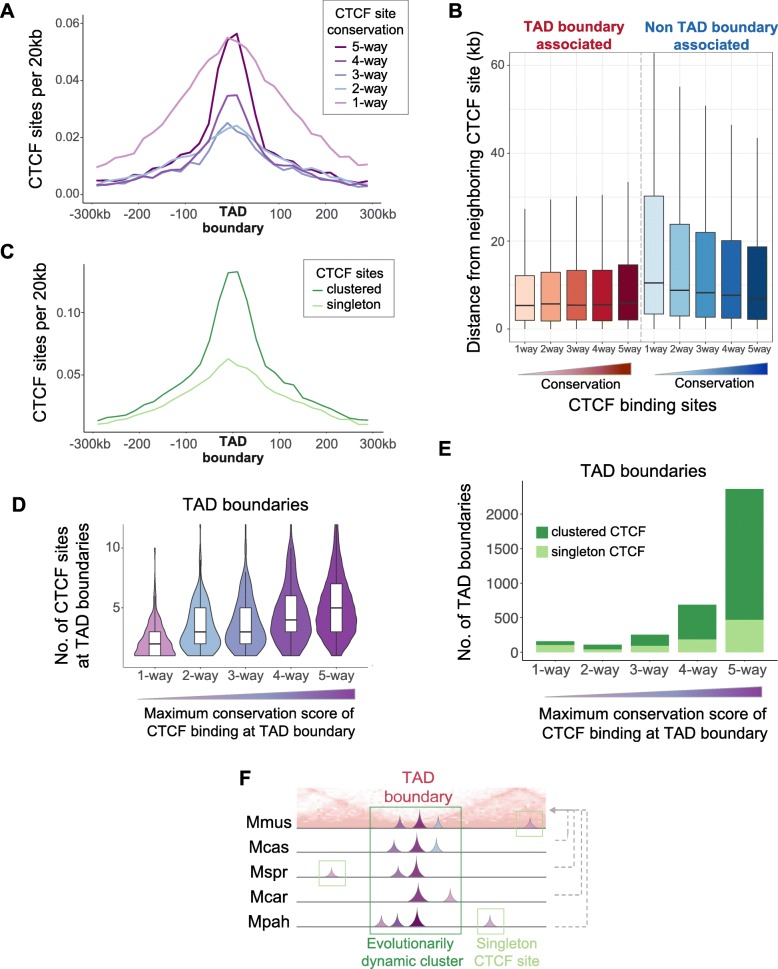
TAD boundaries harbor clusters of both conserved and divergent CTCF binding sites. **a** Both *Mus*-conserved and species-specific CTCF binding sites are highly enriched around TAD boundaries. CTCF sites shared by two to four species are also enriched around TAD boundaries. **b** TAD boundary-associated sites lie significantly closer to each other compared to non-TAD boundary-associated CTCF sites (Mann-Whitney *U* test: *p* < 2.2e−16). **c** CTCF binding sites that belong to a cluster (clustered) are more enriched at TAD boundaries than singleton CTCF sites. **d** The violin plots correspond to TAD boundary regions categorized according to the maximum conservation level of CTCF binding they contain. A TAD boundary region separating two adjacent TADs is defined as the first nucleotide of the downstream TAD ± 50 kb. Each violin plot shows the distribution of the total number of CTCF sites that occur at the TAD boundary regions in the category. TAD boundary regions with at least one *Mus*-conserved site (right-most violin plot) also have a higher number of CTCF sites overall (higher redundancy). In contrast, TAD boundaries that do not contain any species-conserved CTCF sites (left-most violin plot) have much lower numbers of CTCF binding sites. There is a progressive association between the presence of individual conserved CTCF sites with higher abundance of CTCF sites. **e** The bars correspond to TAD boundary regions categorized according to the maximum conservation level of CTCF binding they contain. Dark green demarcates TAD boundaries with clustered CTCF sites; light green shows TAD boundaries with only singleton sites. TAD boundaries that harbor species-conserved CTCF sites also contain CTCF site clusters. **f** Schematic representation of evolutionarily dynamic clusters of CTCF sites that commonly occur at TAD boundaries. TAD borders usually have at least one 5-way conserved CTCF site that is clustered with other sites of lower conservation, including species-specific ones. These CTCF clusters preserve CTCF binding potential at TAD boundaries

To gain further insight into the architecture of TAD boundaries, we investigated the organization of CTCF binding sites within them. In particular, we examined how the density of CTCF binding sites is related to the distance from the TAD boundary. By grouping the CTCF binding sites based on conservation level, we observed that, as expected, TAD borders were highly enriched for conserved CTCF binding events (Fig. 4a). However, species-specific CTCF binding sites were, surprisingly, also enriched at TAD boundaries (Fig. 4a). Thus, TAD boundaries harbor both numerous conserved CTCF binding sites and a high concentration of species-specific CTCF sites. Additionally, TAD boundary-associated sites were consistently close to a neighboring site (median distance ≈ 5.3–5.9 kb) regardless of their conservation level (Fig. 4b). In contrast, CTCF binding sites not associated with a TAD boundary region were further apart from each other (Mann-Whitney *U* test: *p* < 2.2e−16) and the median distance to their closest neighboring site was dependent on conservation level: 7 kb for 5-way conserved sites to 10.5 kb for species-specific sites (Fig. 4b).

We asked whether TAD borders have a specific structure of CTCF sites by investigating potential ancestral clusters from the full set of CTCF binding sites projected to the C57BL/6J genome (*n* = 56,625; Fig. 1c). We defined a CTCF cluster as a group of at least two CTCF binding sites that are each less than 10 kb apart on the genome. After clustering, we found that 23,232 (43%) sites were singletons whereas 32,393 (57%) were part of 11,507 clusters. Interestingly, we observed that the CTCF sites belonging to a cluster were significantly more enriched at TAD borders than singleton CTCF sites (Fig. 4c). This finding strongly implies that clusters of CTCF binding sites are a fundamental architectural structure of TAD boundaries.

**Fig. 5:**
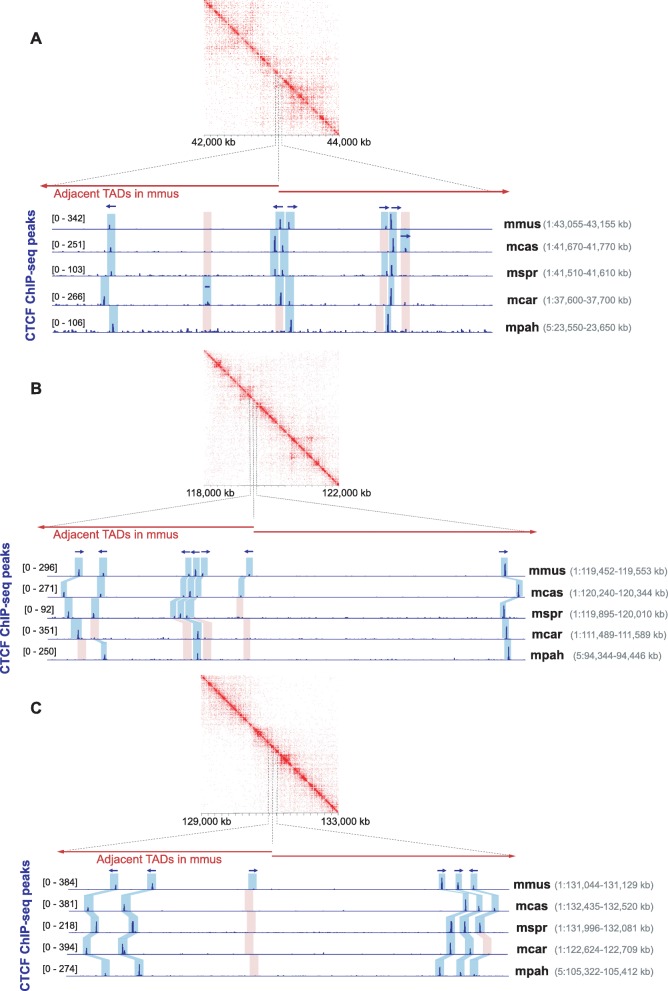
Examples of TAD boundary regions harboring clusters of both conserved and divergent CTCF binding sites. **a**–**c** CTCF ChIP-seq tracks illustrating three examples of TAD boundary regions harboring clusters of closely located CTCF binding sites. Although some of the sites are conserved across species, there are also often lineage-specific gains or losses in the vicinity. Blue shadow boxes highlight the statistically significant peaks identified by MACS, while pink shadow boxes mark CTCF binding losses (orthologous regions with no significant peaks). Arrows indicate the orientations of the CTCF binding motif identified within each peak. In case of more than one motif identified in a peak, the orientation shown corresponds to the motif with the lowest *p* value. The contact maps were visualized using Juicebox [83]

To further characterize the CTCF binding clusters at TAD borders, we asked how features such as redundancy, clustering, and presence of both conserved and non-conserved binding events lying in close proximity are associated with each other. We found that TAD boundary regions with at least one 5-way conserved CTCF site also contained a higher number of CTCF sites overall (Fig. 4d) that mainly belong to clusters (Fig. 4e). This shows that *Mus-*conserved CTCF sites at TAD boundaries usually form clusters with other, more recently evolved CTCF sites (Fig. 4f, Fig. 5).

We questioned whether this phenomenon is solely a characteristic of TAD boundaries or if it is also found in other parts of the genome. We identified 5-way conserved CTCF sites that were not associated with TAD boundaries (selected as *d* > 80 kb from the TAD border to ensure the entire cluster would be *d* > 50 kb) and inspected the CTCF binding profile around them. We observed that additional CTCF sites of various conservation levels, including high numbers of species-specific CTCF sites, were generally accumulated around these *Mus*-conserved sites (Additional file 1: Figure S8). Overall, *Mus-*conserved CTCF binding events are usually part of CTCF binding clusters, rather than appearing as singleton sites. Moreover, although the clusters are apparently stably anchored at 5-way CTCF sites, the cluster as a whole seems to be evolving dynamically, allowing for integration of many evolutionarily younger lineage-specific sites.

We next asked whether clustered CTCF binding sites also have consistent motif orientations by comparing the orientation of lineage-specific gains of CTCF binding sites in a cluster with their neighboring conserved sites. We identified clusters with at least one *Mus-*conserved CTCF site and one gain of a species-specific (1-way) site. Of these clusters, 84.3% include only 5-way CTCF sites with consistent motif orientations and were used to assess whether the newly acquired species-specific CTCF sites had the same orientation as the *Mus-*conserved site(s). A large fraction (70%) of the species-specific gains had the same orientation as all other *Mus-*conserved sites in the same cluster. These newly incorporated sites may have an additive effect in binding or stabilizing CTCF in the region.

Finally, we investigated whether the evolutionary characteristics of clustered CTCF binding across the five species were recapitulated when looking at a single species. We confirmed the enrichment of C57BL/6J CTCF sites of any conservation level at TAD boundaries (Additional file 1: Figure S9A) and that clustered CTCF sites in C57BL/6J were also more highly enriched at TAD boundaries than singleton CTCF sites (Additional file 1: Figure S9B), as observed in all *Mus* species (Fig. 4a, c). Moreover, we found that half of C57BL/6J CTCF binding sites were clustered, similar to the full set of *Mus* CTCF binding regions (Additional file 1: Figure S9C). We also found that the conservation of whole clusters of CTCF sites in C57BL/6J was similar to that of individual CTCF binding sites (Additional file 1: Figure S9D). This implies that clusters of CTCF sites are evolving under selective pressure similar to that underlying the conservation of individual CTCF binding sites.

In summary, clusters of CTCF binding sites of all conservation levels are a common characteristic of TAD boundaries maintained by dynamic evolutionary processes with species-specific sites playing a prominent role. In addition, CTCF clusters with similar characteristics can also be found distant to TAD borders suggesting a broader role in genome function.

### Clusters of CTCF binding sites colocalize with cohesin and regulate gene expression

**Fig. 6:**
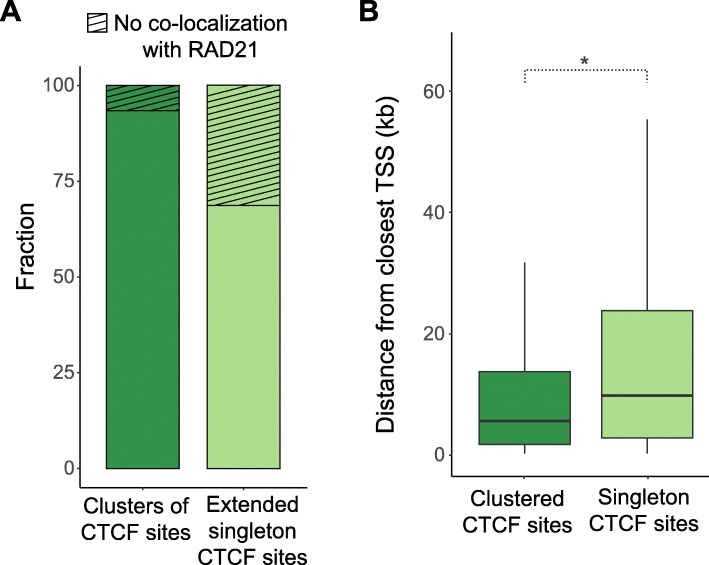
Clustered CTCF sites overlap more frequently with cohesin and locate closer to genes, compared to singleton CTCF binding sites. **a** 93.7% of the clusters of CTCF binding sites demonstrate colocalization with the cohesin subunit RAD21, while the respective fraction of extended singleton CTCF sites is 69% (*χ*^2^ test: *p* < 2.2e−16). The singleton CTCF binding regions were extended by a few kilobases prior to intersection with RAD21 binding regions to ensure the mean of their length distribution is equal to the mean length distribution of clusters of CTCF sites. **b** CTCF sites that belong to clusters (clustered) are located closer to gene TSSs (median distance = 5.3 kb) than singleton CTCF sites (median distance = 10.9 kb) (Mann-Whitney *U* test: *p* < 2.2e−16)

To gain further insight into possible additional functional roles of CTCF binding site clusters, we performed ChIP-seq for the cohesin subunit RAD21 in C57BL/6J. CTCF is known to interact with cohesin to form chromatin loops [20, 45–49]. To control for the longer genomic regions spanned by CTCF clusters, we extended the genomic intervals around the singleton CTCF sites such that the mean of their length distribution was equal to that of the CTCF site clusters (Additional file 1: Figure S10). We found that CTCF site clusters were significantly more likely to overlap with regions enriched for RAD21; 93% compared with only 69% for singleton CTCF sites (*χ*^2^ test, *p* < 2.2e−16) (Fig. 6a). This suggests that clusters of closely located CTCF binding sites help stabilize cohesin and may represent anchors of chromatin loops or TAD boundaries.

CTCF is also known to bind near gene promoters [50]. We measured the distance of each CTCF site belonging to a cluster to the nearest transcription start site (TSS) and compared this distribution to the corresponding distances for singleton CTCF sites. We found that CTCF sites belonging to a cluster are generally located significantly closer to TSSs (median distance = 5.3 kb) than singleton CTCF sites (median distance = 10.9 kb) (Mann-Whitney *U* test, *p* < 2.2e−16; Fig. 6b) which suggests that clusters of CTCF sites may also play an integral role in regulating gene expression.

### Species-specific losses of conserved binding events at TAD boundaries have no detectable impact on local gene expression patterns

CTCF binding sites at TAD boundaries are thought to enhance contact insulation between regulatory elements of adjacent TADs [7], and therefore, their disruption can lead to local ectopic interactions between promoters and enhancers [5, 24, 29]. However, the impact of such disruptions on local gene expression has not been systematically investigated. Here, we took advantage of natural genetic variation in closely related mouse species and our own CTCF binding data to study the effect of CTCF binding site loss in a model fixed by evolution. This approach offers significant advantages over many other experimental approaches, such as disruption of specific CTCF sites [5, 24, 25, 27], haploinsufficiency models [51], or transient acute depletion systems [30–32] in which there is a global disruption of cellular equilibrium.

**Fig. 7:**
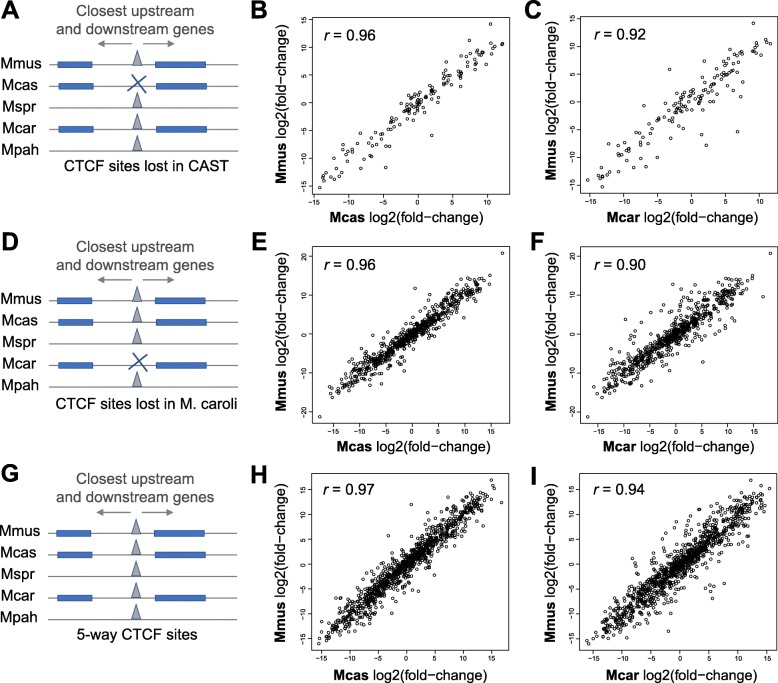
Gene expression patterns around TAD boundaries are robust to local species-specific losses of individual CTCF sites. **a** We identified *M. musculus castaneus* (CAST)*-*specific CTCF site losses at TAD boundaries and estimated the gene expression patterns around them, by calculating the log_2_(fold change) between the closest downstream to the closest upstream gene. **b**, **c** Comparisons of log_2_(fold change) values of gene pairs flanking the CAST-specific losses of CTCF sites between C57BL/6J and CAST, with inconsistent CTCF binding, as well as between C57BL/6J and *M. caroli*, with consistent CTCF binding. Only genes that have a one-to-one orthologous relationship and similar gene lengths among C57BL/6J, CAST, and *M. caroli* were used*.* **d** *M. caroli-*specific CTCF site losses at TAD boundaries and estimated the gene expression patterns around them, with calculated log_2_(fold change) between the closest downstream and the closest upstream gene. **e**, **f** Comparisons of log_2_(fold change) values of gene pairs flanking the *M. caroli*-specific losses of CTCF sites between C57BL/6J and CAST, with consistent CTCF binding, as well as between C57BL/6J and *M. caroli*, with inconsistent CTCF binding. **g** For reference, *Mus-*conserved CTCF sites and calculated gene expression patterns around them with computed log_2_(fold change) of the closest downstream to the closest upstream gene in each of the species. **h**, **i** Comparisons of log2(fold change) values of gene pairs flanking the examined *Mus-*conserved CTCF sites between C57BL/6J and CAST, as well as between C57BL/6J and *M. caroli*

We investigated the instances at TAD boundary regions where a CTCF binding event was conserved in all but one of the five study species. We estimated the impact of these changes on the expression of proximal genes using RNA sequencing (RNA-seq) in C57BL/6J, CAST, and *M. caroli*. First, we identified either CAST-specific (Fig. 7a) or *M. caroli-*specific losses of individual CTCF binding events at TAD boundaries (Fig. 7d). For each of these lost CTCF sites, we found the closest upstream and the closest downstream one-to-one orthologous gene in all three species (Fig. 7a, d) and calculated the relative gene expression of this gene pair (expressed as *log*_*2*_ *fold change*) in each of the species (see the “Methods” section). We then compared these relative expression patterns among the three species.

We found no impact on local gene expression patterns due to species-specific losses of individual CTCF binding events at TAD borders (Fig. 7b, c, e, f, h, i). This suggests that expression patterns of genes at the borders of TADs are robust to the losses of individual CTCF binding even in cases where the binding event is preserved in multiple other closely related species. We propose that the observed CTCF clusters, which may function interchangeably or additively, contribute to the maintenance of this functional resilience.

## Discussion

We used the natural genetic variation of five closely related species to investigate and characterize features of CTCF binding at TAD boundaries. Our analyses reveal that CTCF binding sites at the boundaries of TADs are generally subject to stronger sequence constraints compared to CTCF sites in the background genome. Nevertheless, the CTCF binding profile at TAD borders seems to also be evolving under the effect of dynamic evolutionary processes. This is indicated by numerous gains of new species-specific CTCF binding sites close to species-conserved ones, giving rise to mixed clusters containing both evolutionarily old and young CTCF binding sites.

Our data show that CTCF binding is largely conserved across *Mus* species, consistent with prior studies that demonstrate conservation across mammals [34, 40, 41]. Our data also indicate that the boundaries of TADs commonly overlap with *Mus*-conserved CTCF sites, similar to observations from more distantly related mammalian lineages [10, 37]. We show that a significant fraction of species-specific CTCF sites also localizes in the vicinity of TAD borders and that CTCF binding sites at TAD boundaries have both stronger sequence constraints and stronger binding affinity, independent of their conservation across species. Our data also reveal discrepancies in the expansion of TE classes at TAD boundary regions compared to the background genome. Specifically, TAD boundaries are relatively depleted of both LINE elements and LINE-derived CTCF binding sites, suggesting negative selection against insertions of long—and potentially disrupting—sequences at TAD boundaries. This is complementary to observed structural variant depletion at TAD boundaries as an effect of purifying selection [44]. Overall, these observations suggest that the functional role of CTCF binding at TAD boundary regions is maintained by multiple evolutionary mechanisms including local sequence constraint, new site acquisition, and rejection of insertions and deletions.

Our results show that dynamically conserved regions that contain clusters of CTCF sites are another common characteristic of TAD boundaries. These clusters comprise both conserved CTCF binding events, which were apparently fixed at TAD boundary regions in the common ancestor, and divergent sites, which are the result of more recent gains or losses within the distinct mouse lineages. The conservation is exemplified by genomic regions with CTCF clusters in one species also usually harboring clusters in orthologous *Mus* genomic regions. However, the dynamic nature of these clusters is reflected in the number of sites contained in the cluster which can slightly vary among species, due to gains or losses of individual binding sites. These clusters suggest a mechanism by which local turnover events can largely preserve TAD structure and function. Indeed, a recent study has demonstrated CTCF binding site turnover at loop anchors mediated by TEs, and it suggested that this is a common mechanism of contributing to conserved genome folding events between humans and mice [52]. Based on these observations, we conclude that the formation of CTCF binding site clusters serves as an additional evolutionary buffering mechanism to preserve the CTCF binding potential of TAD boundaries and ensure the resilience of higher-order chromatin structure by maintaining a dynamic redundancy of CTCF binding sites.

In light of two recent studies on the effective occupancy of CTCF sites across the genome, it seems likely that our observations of CTCF binding clusters and increased binding affinity of individual CTCF sites at TAD boundaries are a mechanism to ensure higher effective CTCF occupancy at TAD boundary regions. In particular, Holtzman et al. have provided evidence that not all CTCF binding sites are occupied simultaneously [53]. Furthermore, based on the observed variance of CTCF ChIP-seq read count at different sites, Cattoglio et al. have suggested that some CTCF binding sites are occupied most of the time, while other sites are rather rarely bound by CTCF [54]. Considering these reports, our observations of higher CTCF binding affinity—as predicted by motif sequence and confirmed by ChIP enrichment signal and read coverage—at TAD boundaries, as well as the occurrence of clusters of closely located CTCF sites, are very likely mechanisms that ensure a higher “time-averaged occupancy” of these regions by CTCF. This could increase the chances of a CTCF molecule being engaged at these specific sites when cohesin extrudes chromatin.

Evolutionarily conserved clusters of CTCF binding sites may also help explain previous observations of TAD structures remaining intact upon experimental disruption of individual or multiple CTCF sites, assuming that such clustered CTCF binding sites can be used interchangeably to provide higher-order resilience against local disruptions. For example, Nora et al. showed that the deletion of a TAD boundary is followed by ectopic *cis*-interactions locally but adjacent TADs do not merge; they hypothesize that there must be additional elements within TADs that “act as relays when the main boundary is removed” [5]. Furthermore, Barutcu et al. demonstrated that TAD structures are preserved upon deletion of the CTCF-rich *Firre* locus from a TAD boundary [25]. They hypothesize that additional CTCF binding sites outside the *Firre* locus may serve to recruit CTCF and thus help maintain the TAD boundary. In addition, a recent study on CTCF hemizygosity suggested that, within genes, adjacent CTCF sites may have subtle additive effects on gene expression [55], suggesting that clustered CTCF sites may enhance other CTCF functions. We also found that gene expression around TAD boundaries in cases of species-specific losses of individual CTCF sites is highly robust. As a whole, our results strongly suggest that the dynamic conservation of genomic regions harboring clusters of CTCF sites is an important feature of CTCF binding evolution, which is critical to the functional stability of higher-order chromatin structure. Interestingly, such clusters are also found in genomic regions other than TAD borders. It is possible that these regions are related to the establishment of higher-order chromatin structure, potentially representing unidentified TAD boundaries or loop anchors, or other functional and regulatory roles of CTCF.

Further insight into the functional implications of CTCF site clusters come from our result that CTCF clusters colocalize with the cohesin subunit RAD21 to a greater frequency than singleton CTCF sites. Moreover, we demonstrate that clustered CTCF sites are located significantly closer to TSSs than singleton sites. Together, these suggest that clusters play an important role in stabilizing cohesin at specific genomic regions, as well as in transcriptional regulation. These observations may provide new mechanistic insight to the previously proposed dynamic loop maintenance complex (LMC) model, in which cohesin associates with a genomic region for a significantly longer time than CTCF molecules [56]. Specifically, our observations of clustered CTCF binding sites support the proposed rapid unloading and rebinding of CTCF molecules in close genomic proximity, which facilitates rapid cohesin translocation on DNA between CTCF binding sites that act as occasionally permeable boundary elements [56, 57]. This process apparently facilitates gene transcription by allowing RNA polymerase II to push cohesin along gene bodies [57–59].

Finally, it is tempting to speculate a connection between our identified clusters of closely located CTCF binding sites on the genome and the reportedly observed 3D “clusters” (or “hubs”) of CTCF protein molecules [60, 61]. In particular, Hansen et al. have proposed a guided mechanism where an RNA strand can bind to and gather together multiple CTCF protein molecules near cognate binding sites. These CTCF molecule hubs apparently enhance the search for target binding sites, increase the binding rate of CTCF to its related sites (also as part of the LMC model) and are often implicated in chromatin loop formation [60, 61]. It is possible that our identified CTCF site clusters act synergistically with this mechanism as nearby sites for the concentrated CTCF molecules to bind.

## Conclusions

In conclusion, we identified dynamic evolutionary clusters of CTCF binding sites as a feature of TAD boundary architecture, and we propose that these likely contribute to the remarkable resilience of TAD structures and gene expression to losses and gains of individual CTCF binding sites. Thus, further studies seeking a definitive understanding of the functional roles of CTCF might require consideration of extended regions that harbor clusters of multiple CTCF sites.

## Methods

### ChIP-seq experiments and data analysis

To characterize the CTCF binding profile in *Mus musculus castaneus* (CAST/EiJ) and *M. spretus* (SPRET/EiJ), we performed chromatin immunoprecipitation experiments followed by high-throughput sequencing (ChIP-seq) using adult liver tissue. ChIP-seq libraries and input control libraries from three biological replicates of each species were prepared as described in [62]. Subsequently, libraries were sequenced on a HiSeq2000 (Illumina) to produce 100-bp paired-end sequence fragments.

In addition, we obtained published CTCF ChIP-seq data from the livers of *Mus musculus domesticus* (C57BL/6J), *Mus caroli*/EiJ, and *M. pahari*/EiJ [35]. Three biological replicates from each species were used.

We aligned sequenced reads from CAST and *M. spretus* to the reference genome assemblies CAST_EiJ_v1 and SPRET_EiJ_v1 [63], respectively, with BWA mem version 0.7.12 [64] discarding reads with more than three occurrences. We also mapped the retrieved raw ChIP-seq reads from C57BL/6J, *M. caroli*, and *M. pahari* to the genomes GRCm38 (mm10), CAROLI_EIJ_v1.1, and PAHARI_EIJ_v1.1 [63, 65], respectively, using the same method for the sake of performing matched analyses in all species. CTCF enrichment peaks were called with MACS 1.4.2 [66] with a *p* value threshold of 0.001. For downstream analyses, we used peaks identified in at least two replicates of each species (Additional file 1: Table S1). To produce binding heatmaps (Additional file 1: Figure S1B), we used deeptools version 3.3.1 [67]. We first subtracted the appropriate input library from each ChIP sequencing library using the bamCompare tool. Then, for each species, we produced heatmaps corresponding to the number of ChIP reads—input reads within all peaks found in at least two replicates using the computeMatrix and plotHeatmap tools.

We also performed ChIP-seq in C57BL/6J liver to identify genomic regions enriched for the cohesin subunit RAD21, using also an input control library from C57BL/6J liver from Thybert et al. [35]. Sample preparation and chromatin immunoprecipitation was performed as described in Schmidt et al. [34] using 10 μg RAD21 antibody (Abcam, ab992, lot GR12688-8). Immunoprecipitated DNA and 50 ng of input DNA were used for library preparation using the ThruPLEX DNA-Seq library preparation protocol (Rubicon Genomics, UK). Library fragment size was determined using a 2100 Bioanalyzer (Agilent). Libraries were quantified by qPCR (Kapa Biosystems). Pooled libraries were deeply sequenced on a HiSeq2500 (Illumina) according to the manufacturer’s instructions to produce single-end 50-bp reads. We obtained sequenced reads and mapped them to the mouse genome assembly GRCm38 using BWA 0.6.1 [64]. We then called RAD21 peaks using MACS2 2.1 with default options [66].

### TADs

We used the boundaries of mouse liver TADs published by Vietri Rudan et al. [15]. We considered TAD boundaries as the start and end nucleotides of each TAD, while in some of the analyses (where indicated in the following method description), we used a window of ± 50 kb around them to study TAD boundary regions.

### Conservation of CTCF binding sites in *Mus* species

To investigate the conservation of CTCF binding across the studied *Mus* species, we first found the orthologous alignments of the CTCF ChIP-seq peaks in the genomes of the other species. These orthologous CTCF regions across mice were obtained using an extended version of the eutherian mammal Endo-Pecan-Ortheus (EPO) multiple genome alignment that also included the genomes of CAST, *M. spretus*, *M. caroli*, and *M. pahari* [35]. Once the orthologous regions of CTCF sites were identified in all *Mus* species, we cross-validated the binding of CTCF in each species using the corresponding ChIP-seq data. Specifically, we considered that a CTCF site was conserved if it (a) had an orthologous alignment across species and (b) the orthologous alignments also contained a CTCF ChIP-seq peak (Fig. 1c).

### Binding affinity and sequence constraint of CTCF motifs

To identify CTCF binding motifs, we retrieved the FASTA sequences of all CTCF peaks in C57BL/6J, using bedtools getfasta v.2.25.0 [68], and scanned these sequences for the primary CTCF binding motif (M1) from the JASPAR database [69] using Find Individual Motif Occurrences (FIMO) from the MEME suite v.4.12.0 [70, 71] with default parameters. We extended the identified 19 base-long M1 motifs to include 20 bases upstream and 20 bases downstream in order to allow the discovery of the extended version of the motifs (M1 and M2). Finally, we calculated the binding affinity of these sequences for CTCF using DeepBind v.0.11 [72], as in Aitken et al. [55], and compared the significance of the difference between distributions of the affinity values between motifs found in TAD boundary-associated and non-TAD boundary-associated CTCF peaks at each conservation level (Fig. 2a, b).

To retrieve rejected substitution (RS) scores for each position of every identified 19 base-long M1 motif in C57BL/6J, we obtained pre-calculated GERP [42] conservation scores for each nucleotide of these mouse M1 sequences from Ensembl [73]. The RS score of a genomic position was calculated as the difference of observed to expected substitutions. We then averaged the RS score per position among all motifs and compared these averaged RS scores of TAD boundary-associated M1 motifs with non-TAD boundary-associated motifs (Fig. 2e, f).

### ChIP-seq enrichment and read coverage of identified CTCF peaks

The CTCF sites that we identified in each species were the intersection of the CTCF peaks called in ≥ 2 biological replicates. We calculated the ChIP-seq fragment enrichment of each CTCF site by averaging the ChIP enrichment scores, reported by MACS, over the replicates. We then compared the significance of the difference between the distributions of average ChIP enrichment between TAD boundary-associated and non-TAD boundary-associated CTCF sites of each conservation level using Mann-Whitney *U* tests (Fig. 2c, d).

We used bedtools multicov v.2.25.0 to calculate the counts of read alignments at TAD boundary-associated versus non-TAD boundary-associated CTCF peak regions, in a total of five C57BL/6J replicates (Additional file 1: Figure S6). To increase the robustness of our observations, we added two additional replicates to the three initial ones, which we processed in the same way as the other replicates (see the “ChIP-seq experiments and data analysis” section).

### Motif word usage analysis

We scanned all CTCF peaks from each of the five species for the primary CTCF binding motif (M1) using FIMO from the MEME suite as described above. From the 19 base M1 motif instances identified in each species, we retrieved the central most informative 14-mer and estimated its frequency of occurrence as the number of occurrences of the 14-mer word in CTCF binding regions divided by the number of occurrences of the word in the whole genome of the species using the procedure of Schmidt et al. [34]. We filtered out any motif word that occurred fewer than five times in the whole genome. We illustrated the occurrence frequency of the motif words in each species on a heatmap which is sorted by distance to the closest TAD border (Additional file 1: Figure S7).

### Association of CTCF binding sites with classes of transposable elements

We used the full set of CTCF sites identified in all species and projected them on to the C57BL/6J genome (GRCm38), as well as published transposable elements in C57BL/6J (Thybert et al. [35]; https://www.ebi.ac.uk/research/flicek/publications/FOG21). We intersected the center of each CTCF binding site with the transposable elements and reported the number of CTCF site centers that overlapped with each TE class. The overall representation of each TE class in the whole genome that is shown as a reference (marked as “background” in Fig. 3a) was calculated as the total length of all TEs belonging to each class (SINE, LINE, LTR, DNA) sequences divided by the total genome length.

### Representation of TE classes at TAD boundary regions

As for Fig. 3b, we defined TAD boundary regions as genomic windows of 50 kb upstream and 50 kb downstream of the boundaries of TADs. To evaluate the representation of each TE class, we summed the length of sequences corresponding to each TE class that occurred within each TAD boundary region and divided that by the total length of the TAD boundary region, i.e., 100 kb. To retrieve random genomic regions of similar length and distribution, we shuffled the TAD boundary regions using bedtools shuffle v2.2.5.0, having first excluded chromosome Y, genome scaffolds, and chromosome ends, where TADs are not called. We repeated the same calculation for TE class representation as above for these shuffled TAD boundaries, i.e., random genomic regions. We then plotted the distribution of these values for TAD boundary regions and random genomic regions. To determine the representation of each TE class in the background genome (dotted line in Fig. 3b), we divided again the total length of all sequences that correspond to each TE class by the total C57BL/6J genome (GRCm38) length, analogous to the CTCF TE class analysis above.

### Density of CTCF sites at TAD boundaries and clusters of CTCF binding sites

To determine the enrichment of CTCF binding sites in TAD boundary regions (compared to the surrounding genome), we measured the distance of each CTCF binding site to its closest TAD boundary using bedtools closest. We then categorized the CTCF sites based on their conservation level. For each CTCF site conservation level, we grouped all distance values up to ± 300 kb in bins of 20 kb and plotted the number of CTCF sites in each bin divided by the length of the bin, i.e., 20 kb (Fig. 4a). To further characterize the density of CTCF sites at TAD boundaries, we grouped CTCF sites both according to their conservation level and association with a TAD boundary (versus no association with any TAD boundary), and for each of these categories, we found the distance of each CTCF site from its closest CTCF site using bedtools closest (Fig. 4b).

To identify clusters of CTCF binding sites, we used the full set of CTCF binding sites of all five *Mus* species projected onto the C57BL/6J genome (GRCm38/mm10), as shown in Fig. 1c. We identified instances of consecutive CTCF sites that were up to 10 kb apart from each other, using bedtools cluster. We then determined and compared the enrichment of clustered and singleton CTCF sites at TAD boundaries using the same approach as in Fig. 4a but having categorized the CTCF sites based on whether they belong to a cluster (clustered) or not (singletons) (Fig. 4c).

For Fig. 4d, e, we again defined TAD boundary regions as TAD boundary ± 50 kb. We categorized these regions based on the *highest* conservation level of their CTCF sites. Subsequently, for each category, we counted its total number of CTCF sites (Fig. 4d), as well as the number of these TAD boundary regions with clustered CTCF sites and with only singleton sites (Fig. 4e).

For Additional file 1: Figure S8, we defined *Mus-*conserved (5-way) CTCF sites with a distance to the closest TAD border > 80 kb as non-TAD boundary associated. We calculated the enrichment of 1-way (species-specific), 2-way, 3-way, and 4-way conserved CTCF sites in their vicinity in the same way as in for TAD boundaries (Fig. 4a), but using as anchor the non-TAD boundary-associated 5-way CTCF sites themselves, instead of the TAD boundaries.

### Clusters in C57BL/6J and cluster conservation analyses

We identified clusters of CTCF binding sites in C57BL/6J (Additional file 1: Figure S9) in the same way as for Fig. 4c but using only CTCF peaks called in C57BL/6J. We used the same methods as for Fig. 4a, c to determine the enrichment of CTCF sites of different conservation levels at TAD borders (Additional file 1: Figure S9A), as well as the enrichment of clustered versus singleton CTCF sites (Additional file 1: Figure S9B).

To estimate the conservation of CTCF sites clusters (Additional file 1: Figure S9D), we identified all the genomic regions that correspond to clusters of CTCF sites in each of the five species separately. We then projected through whole-genome alignments (see the “Conservation of CTCF binding sites in *Mus* species” section) the cluster regions of each species onto the C57BL/6J genome and determined whether they overlap with the orthologous cluster regions of the other species.

### RNA-seq data

We retrieved published liver-derived RNA-seq data from six biological replicates for each of the species C57BL/6J and *M. m. castaneus* [74], as well as from four biological replicates of *M. caroli* [75]*.* To have the same number of replicates in each species, we further generated and sequenced two additional RNA-seq libraries for *M. caroli* following the methods described in Goncalves et al. [74] and Wong et al. [75]. Briefly, total RNA was extracted from two independent liver samples using Qiazol (Qiagen) and DNase treated with DNA-free DNA Removal Kit (Ambion). Polyadenylated mRNA was enriched, directional double-stranded cDNA was generated, fragmented by sonication, and prepared for sequencing. Each of the two libraries was sequenced on an Illumina GAIIx to generate 75-bp paired-end fragments.

### RNA-seq data processing and analysis

Adapter sequences were trimmed off with reaper from the Kraken tool suite [76]. The paired-end RNA-seq reads from each replicate of C57BL/6J, CAST, and *M. caroli* were mapped to the corresponding species' genomes (see the “ChIP-seq experiments and data analysis” section) using STAR 1.5.2 [77] with default settings. Raw reads mapping to annotated genes were counted using htseq-count [78]. We then used the raw read counts to perform differential expression analyses with DESeq2 1.20.0 [79] with default settings.

To determine the gene expression patterns around instances of 5-way conserved CTCF sites and species-specific CTCF site losses at TAD boundaries (Fig. 7a, d, g), we first identified the closest upstream and downstream gene in each species using the gene annotation from Ensembl version 95 [65] and then calculated the relative gene expression of downstream to upstream gene in each species. We were not interested in the relative expression of the gene pair flanking a CTCF site per se, but in whether this ratio for each CTCF site is consistent between species when the in-between CTCF binding separating them changes. For this reason, we only used CTCF sites that were flanked by 1:1 orthologous genes between the three species. We went on to use DESeq2 [79] in order to compute the log_2_(fold change) between the downstream and upstream gene—as a measure of the relative expression of genes flanking each CTCF site—in each species and to subsequently compare this log_2_(fold change) between species. Since DESeq2 is not designed to normalize for gene lengths, and our aim was to generate comparable expression pattern estimations between the species, we also required all the orthologous genes that we used to have a similar length among the three species (0.7 < *len_ratio* < 1.3, where *len_ratio* is the length of gene in species A divided by the length of its orthologous gene in species B). Finally, we compared the calculated log2(fold change) values for each gene pair in C57BL/6J with the corresponding value of its orthologous gene pair in CAST (Fig. 7b, e, h) and in *M. caroli* (Fig. 7c, f, i).

## Supplementary information

**Additional file 1.** Supplementary figures and table.

**Additional file 2.** Review history.

## Data Availability

All ChIP-seq and RNA-seq data generated in this study are available in the Array Express repository (https://www.ebi.ac.uk/arrayexpress/) under the accession numbers E-MTAB-8014 [80], E-MTAB-8471 [81], and E-MTAB-8016 [82]. Additional ChIP-seq and RNA-seq data that were used in the study are available under the accession number E-MTAB-5769 [35] (ChIP-seq), E-MTAB-1091 [74] and E-MTAB-2483 [75] (RNA-seq). Hi-C-derived TADs and transposable elements were retrieved from Vietri Rudan et al. 2017 [10] and Thybert et al. [35] (https://www.ebi.ac.uk/research/flicek/publications/FOG21), respectively.
