# Supplementary material for: Clustered CTCF binding is an evolutionary mechanism to maintain topologically associating domains
Source: Genome Biol. 2020 Jan 7;21:5. doi: 10.1186/s13059-019-1894-x (PMC6945661; doi:10.1186/s13059-019-1894-x)
Supplement: Supplementary file 1 — Additional file 1. Supplementary figures and table. [file 13059_2019_1894_MOESM1_ESM.docx]

**Additional file 1:** Supplementary figures and table

**
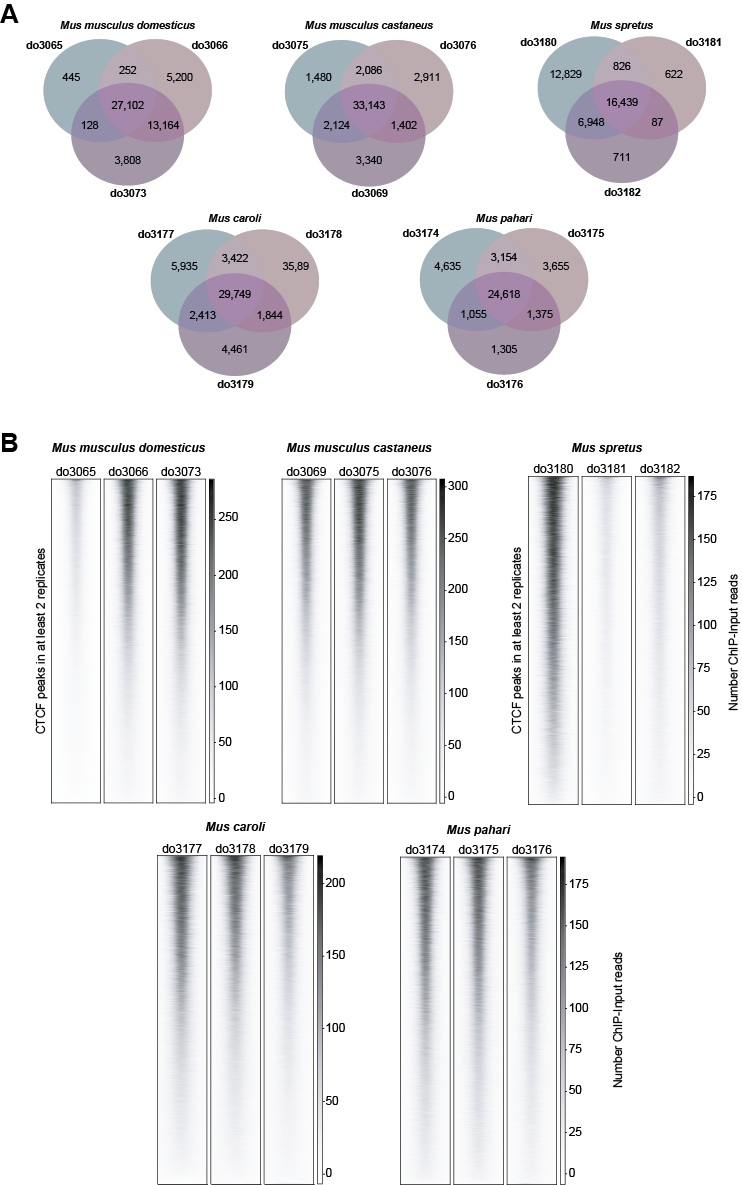
**

**Figure S1**: **CTCF peak reproducibility among replicates of each *Mus* species.** Venn diagrams (A) and binding heatmaps (B) showing reproducibility of peak calling among the three biological replicates from each species. We used peaks that were identified in at least two of the three replicates.


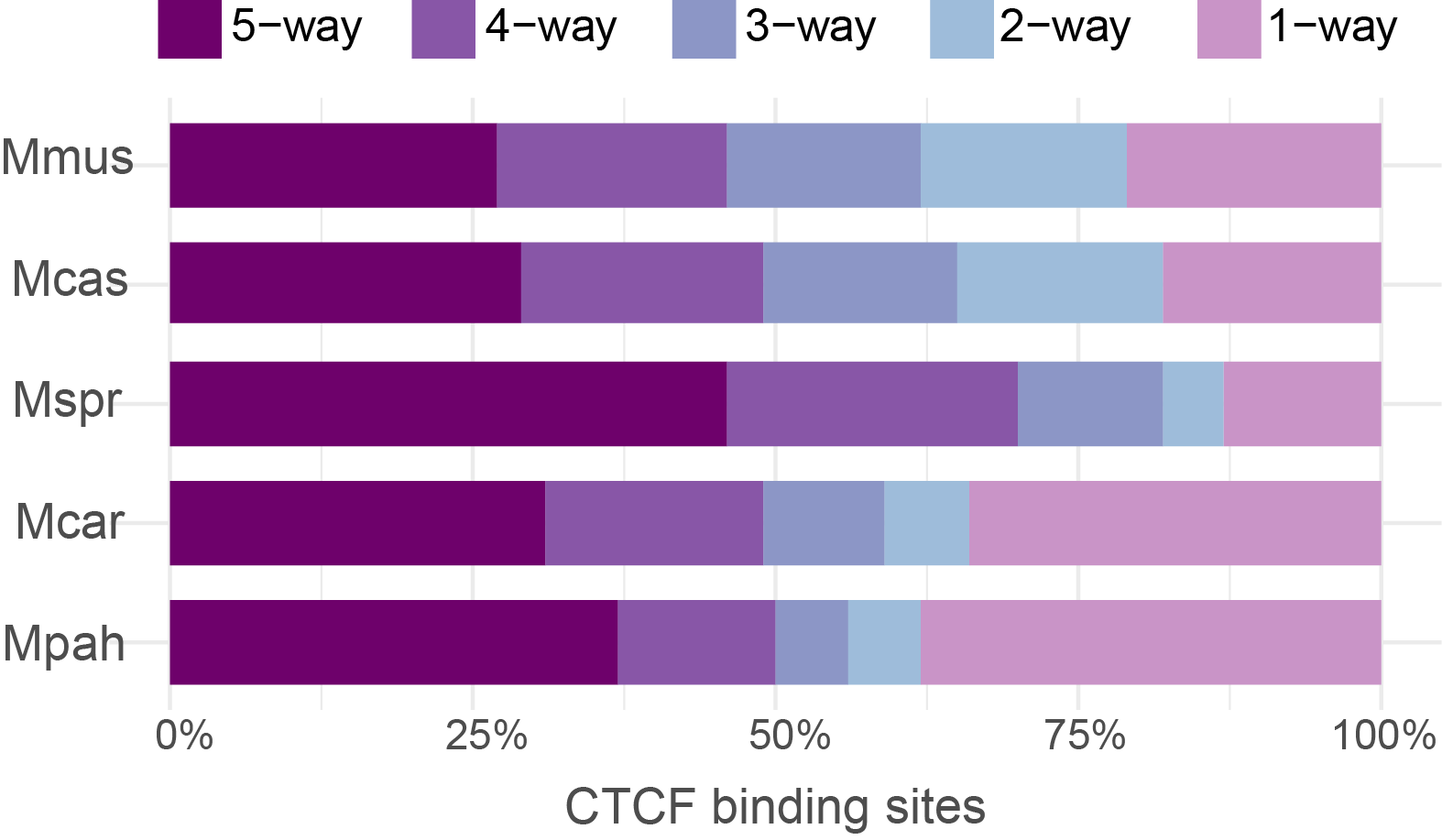


**Figure S2**: **Fractions of CTCF binding sites of different conservation levels in each of the studied *Mus* species.**

**
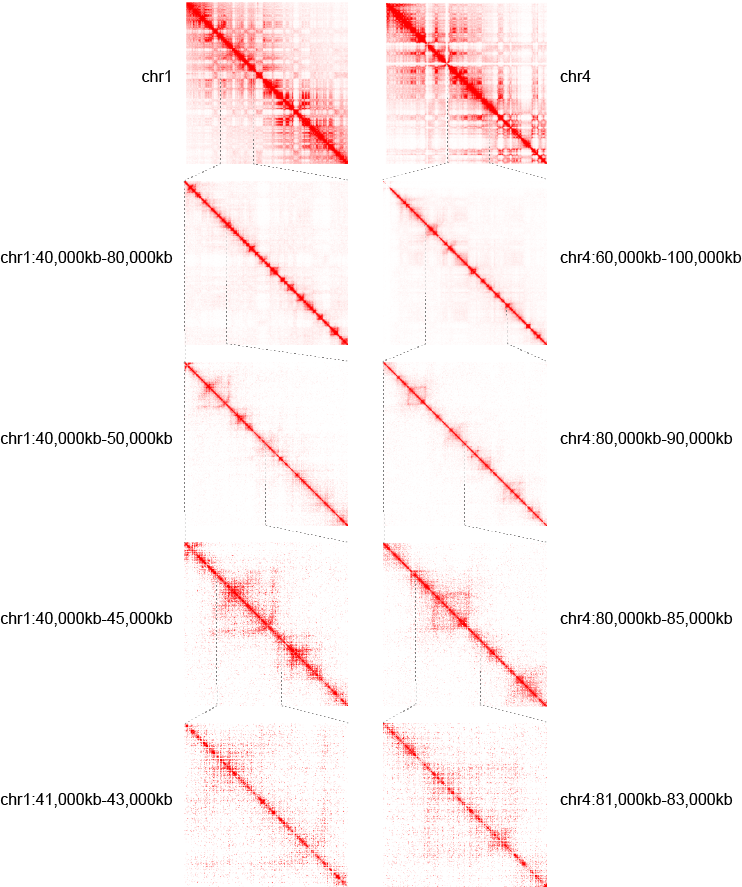
**

**Figure S3**: **Hi-C contact maps from published C57BL/6J liver.**The contact maps generated from the published C57BL/6J Hi-C data [1] were visualized using Juicebox [2]. They show example regions from chromosomes 1 and 4, zoomed at different scales.


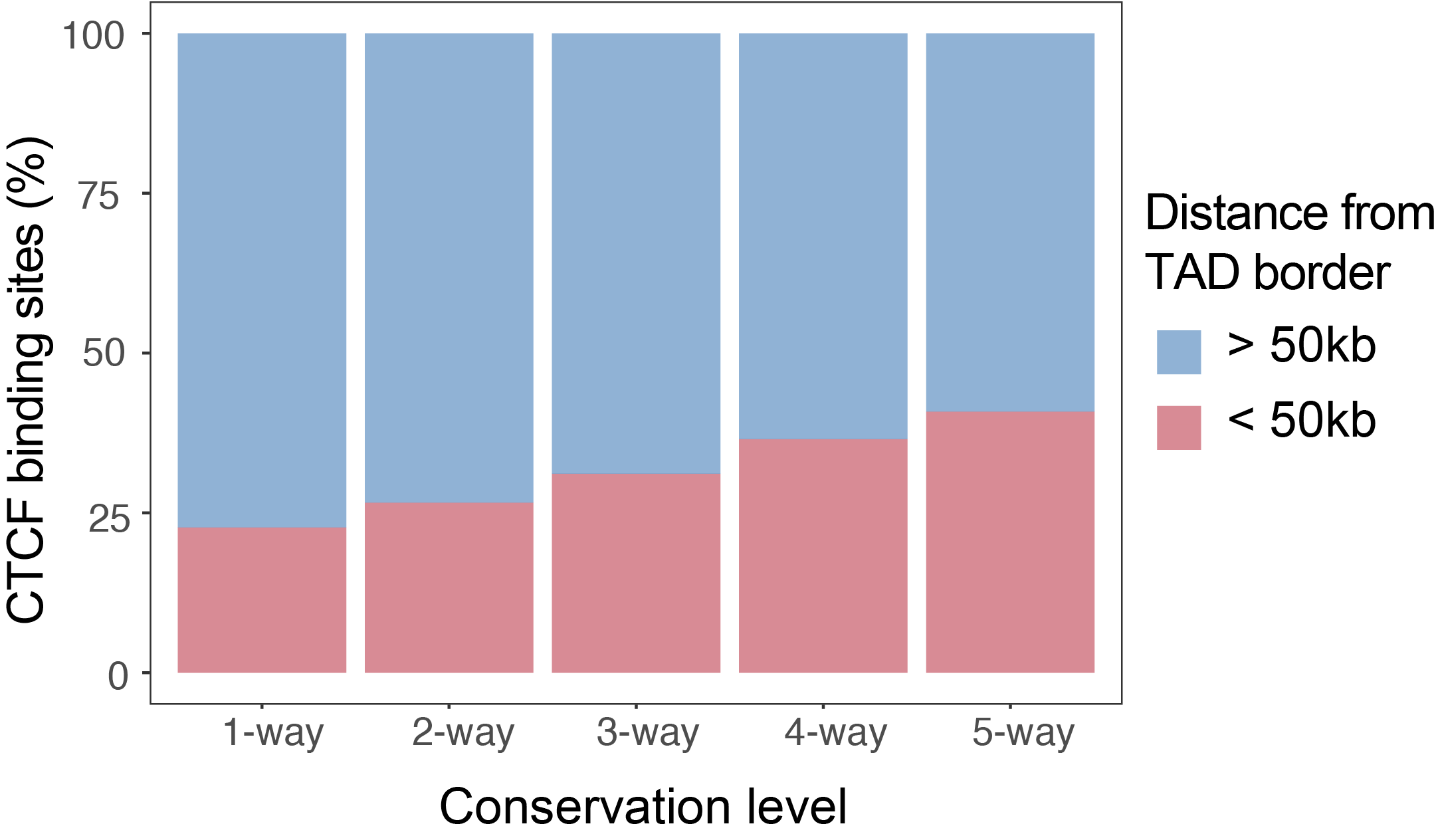


**Figure S4**: **Fractions of all *Mus* CTCF sites of each conservation level that are associated (*d* ≤ 50kb) or not associated (*d* > 50kb) with TAD boundaries.**

**
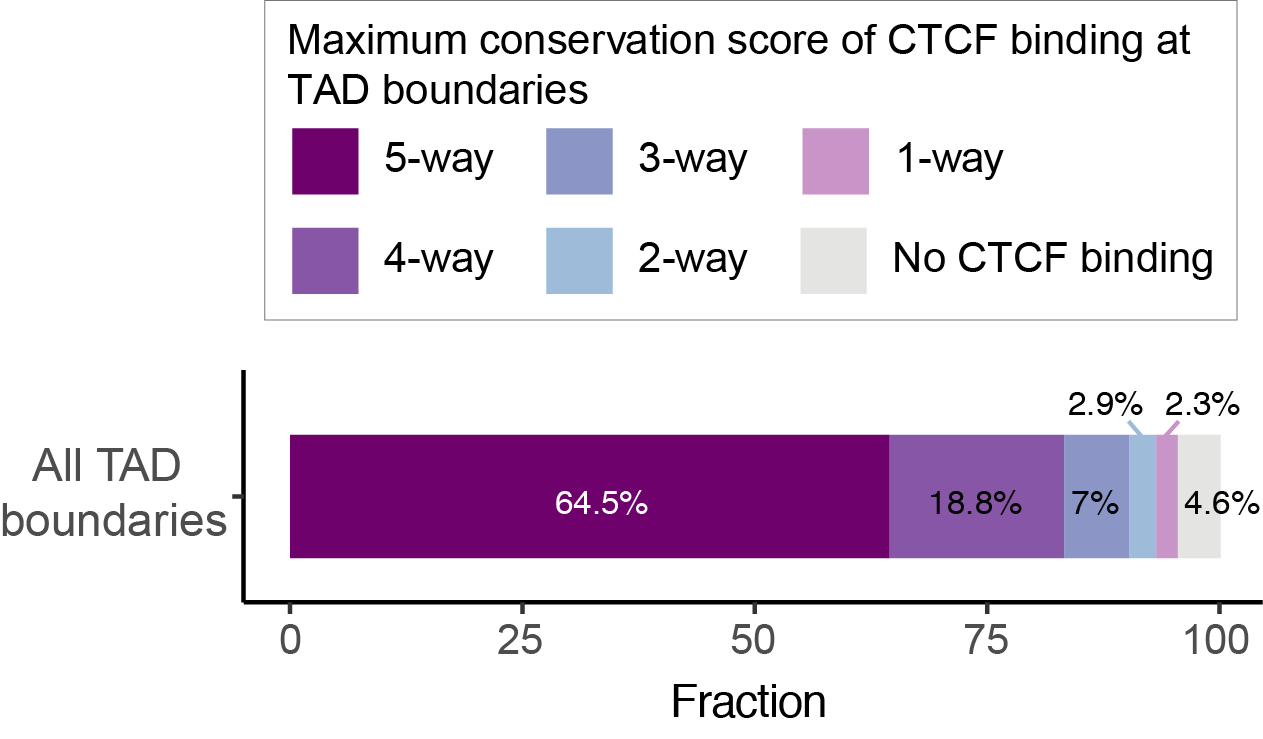
**

**Figure S5**: **Fractions of TAD boundaries with CTCF sites of different conservation levels**. Most TAD boundaries (64%) harbor at least one *Mus*-conserved (5-way) CTCF site. Lower percentages of TAD borders do not contain any *Mus-*conserved CTCF site but overlap with less conserved sites or do not bind CTCF at all.


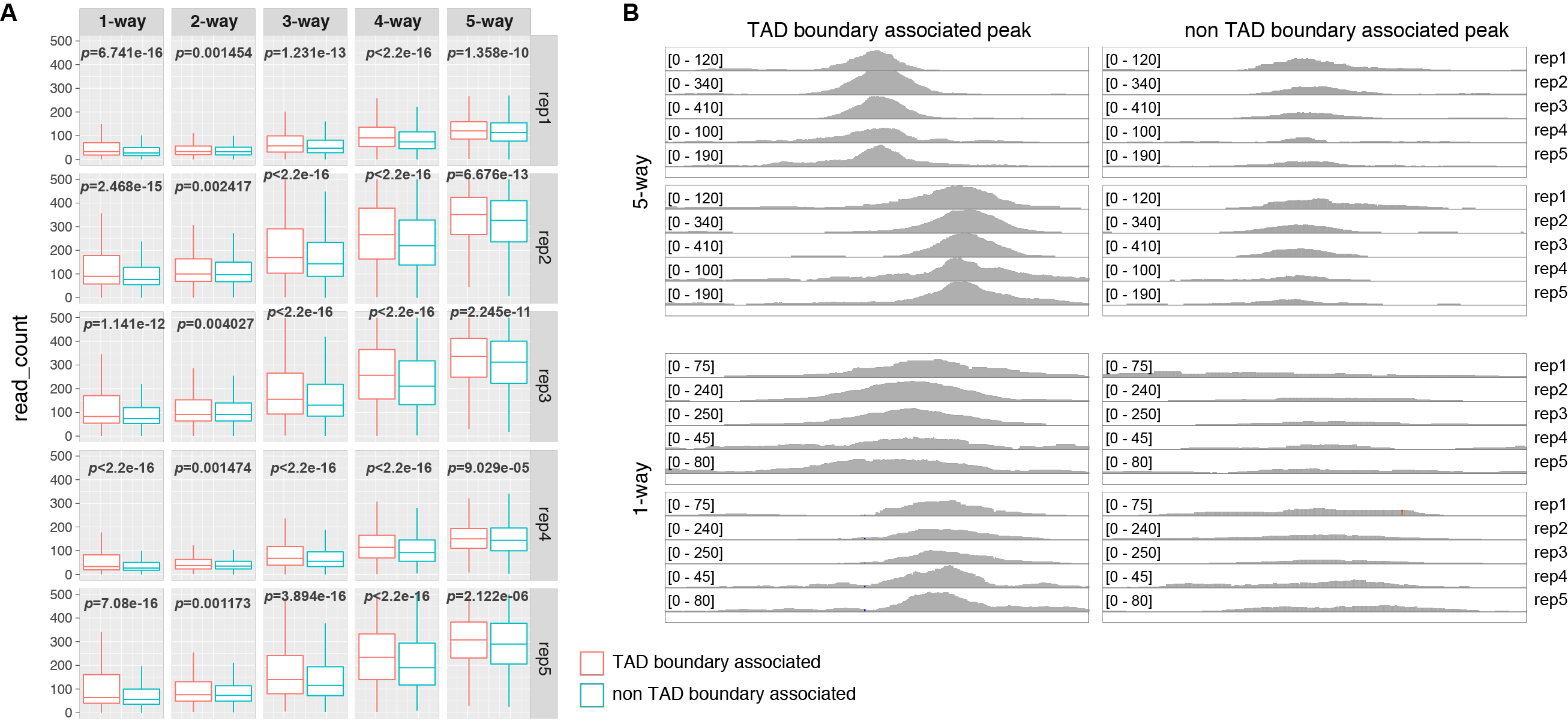


**Figure S6**: **TAD boundary-associated CTCF peaks have higher read coverage compared to non-TAD-boundary-associated peaks.** (A) Mapped read counts for TAD boundary-associated versus non-TAD-boundary-associated peaks in five biological replicates of C57BL/6J liver. The two groups were compared using Mann Whitney *U* tests. (B) ChIP-seq read coverage for example loci of 5-way conserved and species-specific peaks at TAD boundaries (left) compared, respectively, to 5-way and species-specific (1-way) peaks at non boundary regions (right). The read coverage of TAD boundary-associated peaks is higher than at the non-TAD-boundary-associated peaks, independently of whether the peaks are conserved or species-specific. The observations are consistent among the replicates.


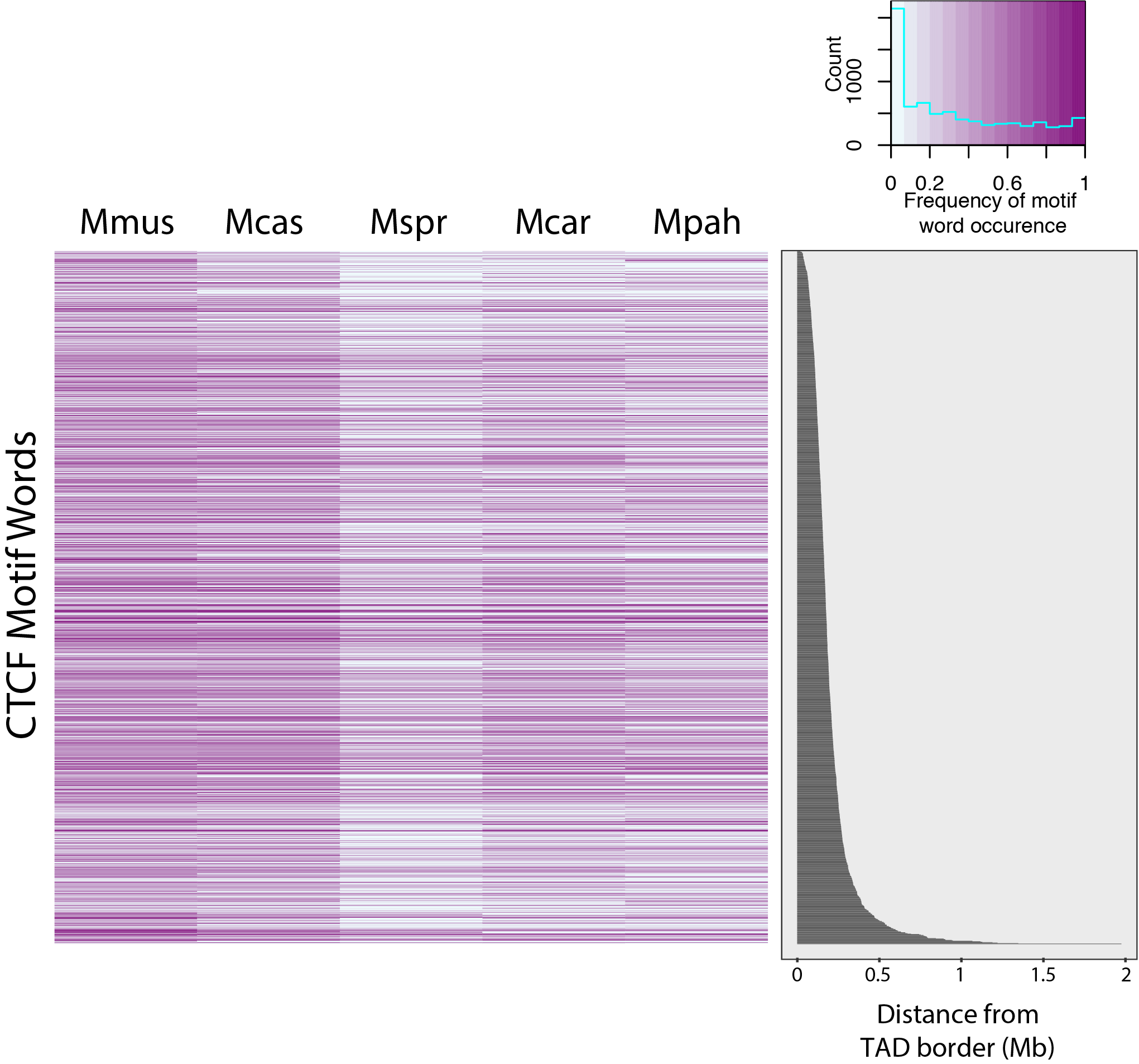


**Figure S7**: **There is no evidence of any enrichment of specific motif words at TAD boundary regions among the species.** Heatmap of the ~1,500 motif words found in CTCF peaks in the five *Mus* species. Each row corresponds to a motif word, while the color density represents its frequency of occurrence. The occurrence frequency of each motif word in the CTCF peaks is normalized by the number of its occurrences in the whole genome for the respective species. Motif words in the heatmap are sorted based on their distance to the closest TAD boundary. There is no evidence of any selected set of motif words being used with significant frequency at TAD boundaries among the species. The lower density of motif words is *M. spretus* reflects the smaller number of CTCF binding sites identified in that species.


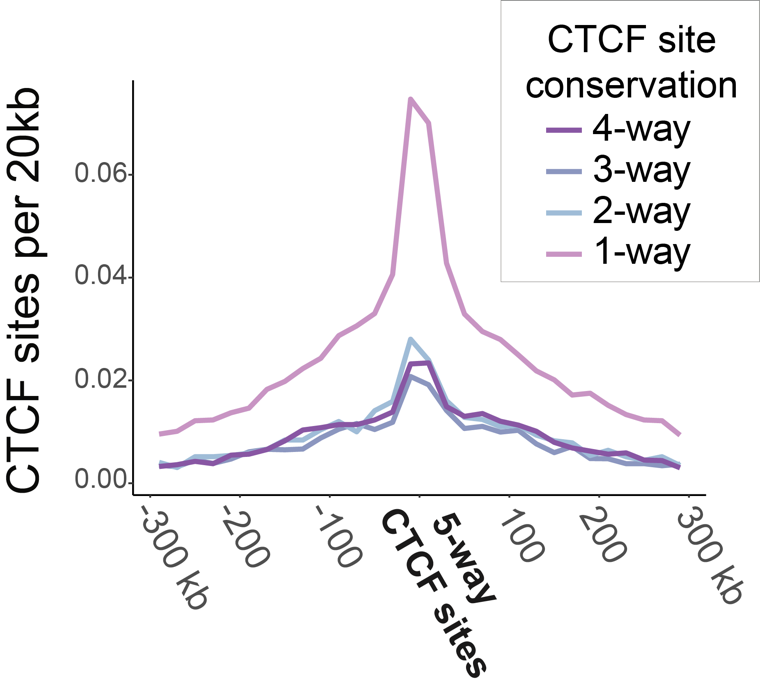


**Figure S8: Clusters of both conserved and species-specific CTCF sites might also occur away from TAD boundaries.** Enrichment of CTCF sites of different conservation levels around *Mus*-conserved CTCF sites that are *not* associated with TAD boundaries (distance from closest TAD border: *d* > 80kb). A high number of species-specific (1-way) CTCF sites are concentrated around these “anchor” 5-way conserved sites, showing that sites of mixed conservation levels can be clustered together.


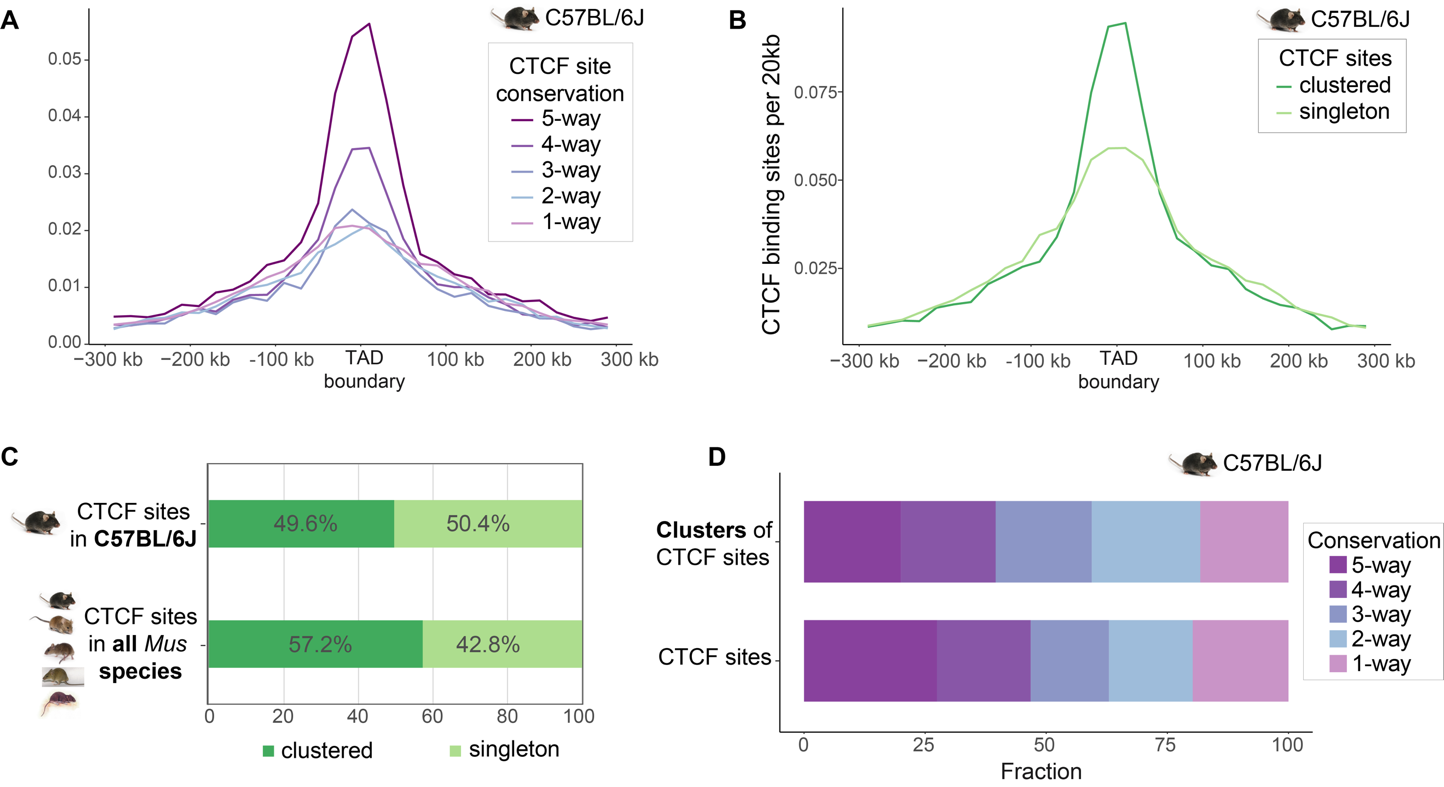


**Figure S9:** **Inspection of the CTCF binding profile in C57BL/6J confirms that CTCF sites form clusters in individual species.** (A) Enrichment of C57BL/6J CTCF sites of different conservation levels at TAD boundaries. (B) Clustered C57BL/6J CTCF sites are more highly enriched than singleton sites at TAD borders. (C) The fraction of clustered CTCF sites in C57BL/6J is similar to that of CTCF sites belonging to ancestral *Mus* clusters. (D) The conservation pattern of CTCF site clusters, as distinct functional entities, resembles that of individual CTCF binding sites.


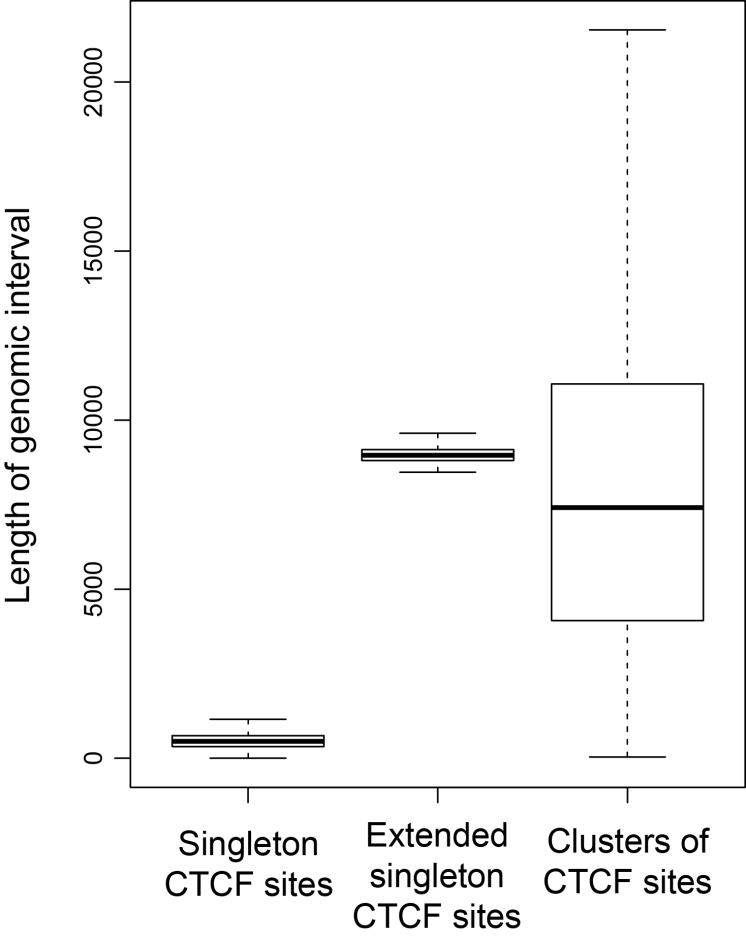


**Figure S10:** **Length distribution of genomic intervals occupied by singleton CTCF sites, “extended” singleton CTCF sites and clusters of CTCF sites.** The extended singleton CTCF sites represent genomic windows of singleton CTCF sites that were extended so that the mean of their length distribution becomes equal to that of the length distribution for the CTCF clusters.

**Table S1:** **Mapping and peak calling statistics for CTCF ChIP-seq data in the five *Mus* species.**

| **Species** | **Factor** | **Individual** | **Unique ID** | **Number Input Reads** | **Number Mapped Reads** | **% Mapped Reads** | **Number of MACS Peaks (per replicate)** | **Number of reproducible peaks (2 replicates)** |
| --- | --- | --- | --- | --- | --- | --- | --- | --- |
| *Mus musculus domesticus* | CTCF | 685304.0 | do3065 | 37,067,110 | 32,106,748 | 87 | 27,927 | 40,289 |
| *Mus musculus domesticus* | CTCF | 685305.0 | do3066 | 63,240,749 | 53,082,610 | 84 | 45,718 |  |
| *Mus musculus domesticus* | CTCF | 85922.0 | do3073 | 49,861,065 | 45,543,182 | 91 | 44,202 |  |
| *Mus musculus domesticus* | input | 85304.0 | do3071 | 46,823,684 | 44,348,418 | 95 | NA | NA |
| *Mus musculus castaneus* | CTCF | 74636.0 | do3075 | 68,333,659 | 65,956,183 | 97 | 38,833 | 38,373 |
| *Mus musculus castaneus* | CTCF | 74874.0 | do3076 | 37,740,031 | 36,120,900 | 96 | 39,542 |  |
| *Mus musculus castaneus* | CTCF | 76993.0 | do3069 | 69,732,659 | 60,847,415 | 87 | 40,009 |  |
| *Mus musculus castaneus* | input | 74636.0 | do3079 | 66,991,879 | 54,675,669 | 82 | NA | NA |
| *Mus spretus* | CTCF | 1a | do3180 | 80,500,930 | 76,032,454 | 94 | 37,042 | 24,183 |
| *Mus spretus* | CTCF | 2c | do3181 | 72,366,179 | 67,433,478 | 93 | 17,974 |  |
| *Mus spretus* | CTCF | 3e | do3182 | 48,076,327 | 44,763,098 | 93 | 24,185 |  |
| *Mus spretus* | input | 3e | do3185 | 30,940,333 | 29,778,137 | 96 | NA | NA |
| *Mus caroli* | CTCF | 76791.0 | do3177 | 84,228,625 | 78,277,357 | 93 | 41,519 | 37,027 |
| *Mus caroli* | CTCF | 76792.0 | do3178 | 75,520,746 | 71,218,023 | 94 | 38,604 |  |
| *Mus caroli* | CTCF | 78713.0 | do3179 | 118,560,403 | 113,114,517 | 95 | 38,467 |  |
| *Mus caroli* | input | 76791.0 | do3184 | 43,083,604 | 41,392,442 | 96 | NA | NA |
| *Mus pahari* | CTCF | 66009.0 | do3174 | 75,214,636 | 66,852,515 | 89 | 33,462 | 29,924 |
| *Mus pahari* | CTCF | 82898.0 | do3176 | 70,433,073 | 64,809,159 | 92 | 28,353 |  |
| *Mus pahari* | CTCF | 82903.0 | do3175 | 70,754,788 | 64,160,019 | 91 | 32,802 |  |
| *Mus pahari* | input | 66009.0 | do3183 | 50,780,061 | 47,658,093 | 94 | NA | NA |

Supplementary references

1. Vietri Rudan M, Barrington C, Henderson S, Ernst C, Odom DT, Tanay A, et al. Comparative Hi-C Reveals that CTCF Underlies Evolution of Chromosomal Domain Architecture. Cell Rep. 2015;10:1297–309.

2. Durand NC, Robinson JT, Shamim MS, Machol I, Mesirov JP, Lander ES, et al. Juicebox Provides a Visualization System for Hi-C Contact Maps with Unlimited Zoom. Cell Syst. [Internet]. 2016;3:99–101. Available from: https://linkinghub.elsevier.com/retrieve/pii/S240547121500054X
