# Supplementary material for: Clustered CTCF binding is an evolutionary mechanism to maintain topologically associating domains
Source: Genome Biol. 2020 Jan 7;21:5. doi: 10.1186/s13059-019-1894-x (PMC6945661; doi:10.1186/s13059-019-1894-x)
Supplement: Supplementary file 2 — Additional file 2. Review history. [file 13059_2019_1894_MOESM2_ESM.docx]

Review History

**First round of review**

**Reviewer 1**

**Are you able to assess all statistics in the manuscript, including the appropriateness of statistical tests used?**

Yes, and I have assessed the statistics in my report.

**Comments to author:**

Review of "Clustered CTCF binding is an evolutionary mechanism to maintain topologically associating domains"

The present paper by Kentepozidou et al. looks at genomics data across five different mouse species to study how variation in CTCF binding affects various functional aspects, such as TADs, gene expression and association with cohesin. I believe the paper is topical and of general interest - CTCF has emerged as the main architectural protein and regulator of 3D genome organization in mammals and is currently under intense interest. A general conundrum in the field has been the fact that whereas almost all TADs are demarcated by CTCF binding sites, most CTCF binding sites do not actually form TADs. The identification of a key role for clustered CTCF binding sites by Kentepozidou et al. offers perhaps a partial solution to this conundrum, which I believe makes it likely that this paper will be of significant interest to the field and beyond.

Recently, there have been a number of perturbation experiments performed where CTCF is acutely depleted and/or specific CTCF binding sites are deleted in the genome and then the effect on 3D genome organization, TADs, gene expression, etc. is assessed. These have been really important and informative. Kentepozidou takes a slightly different approach by exploiting the natural variation between 5 different mouse species. Here, evolution has performed a "natural experiment", which allows the authors to assess how specific CTCF binding sites affect function. The authors are not the first to do this - several previous studies have looked at CTCF across species and/or studied the evolution of CTCF binding sites - but I do think that the present study makes a number of significant and original observations, which go well beyond what has previously been shown.

Finally, as a random tangent, I must say I was quite surprised and intrigued by Figure 4A. I think this is quite an interesting observation.

1. Show me the data please

a. The authors generally do a good job of showing complicated and large datasets and analyses in simple and fairly easy to understand figures. But I think they take it a bit too far. The whole basis of this paper is ChIP-Seq data, but they never actually show us the raw data. I'd like to see a main figure (probably in Figure 1) that shows raw ChIP-Seq tracks (e.g. genome browser) for all 5 mouse species.

I'd also like to set other ChIP-Seq data plots in the supplementary figures additionally.

b. It seems like the authors use the Hi-C data from Vietri Rudan. But this is not Hi-C data for all 5 mouse species and while it is true that TADs are somewhat conserved, some TADs are not. And the fact that the authors do not have high-res Hi-C data for all 5 mouse species is a serious limitation, that I believe that should openly stated in the main text to make this clear to the reader. In fact, I'd like to see the raw Hi-C data that the authors used plotted as contact maps as well to allow the reader to assess the quality.

2. How do the replicates compare?

Since the foundation of this paper is ChIP-Seq data, the quality and reproducibility of this data is key for substantiating the conclusions, but the authors do not actually show this. They say they performed 3 biological replicates. Please show the following:

a. ChIP-Seq tracks for all 5 species and each replicate individually (probably in Supplement).

b. Please perform reproducibility analysis between replicates

c. Please show the binding heatmaps (rows = different CTCF sites, columns = +/- e.g. 2kb) for each replicate individually and also Venn Diagrams between replicates

d. Please include a supplementary figure or table with summary data for all the replicates: number of reads per replicate, number of identified peaks per replicate (e.g. MACS) and other useful summary data

I understand that the raw data is available for download, but I still believe it is essential that the authors perform these analyses and shown them in an easy-to-assess manner in their manuscript.

3. Language

The paper is generally well-written and easy to follow. It is also not too long. But there were a few places where I found the language confusing, including:

Abstract: "Our analyses reveal that CTCF binding is maintained at TAD boundaries by an equilibrium of selective constraints and dynamic evolutionary processes." - can evolution really be said to be in an equilibrium? This implies a thermodynamic stability. I would suggest re-writing or explaining what is meant by an evolutionary equilibrium.

"Dynamic conservation of CTCF site clusters is an apparently important feature …" What does "Dynamic Conservation" mean? Dynamic implies change and conservation implies the opposite of change? Please explain.

Page 19, line 6: "dynamically conserved regions": again it's not very clear to me what "dynamic conservation" means. See also above.

4. Miscellaneous Comments

a. binding affinity. I slightly object to the use of the term "binding affinity" in a purely computational study. I understand that there are ways of algorithmically guestimating these, and I like the fact that the authors did this analysis, but I still think the authors should clearly caveat this and try to avoid using the term "binding affinity" unless they have actually measured it. At least say "computationally estimated binding affinity" or something along those lines.

b. Figure 4D. I was confused about how this was defined. TADs are adjacent and in the simplest model demarcated by convergent CTCF sites. In the simplest model then we would expect there to be at least 2 CTCF sites at the boundary between two TADs; one for the left TAD boundary and one for the right TAD boundary. As such, we should perhaps divide by 2 if we want to quantify the number of CTCF sites per TAD at a TAD boundary.

When the authors report the number of CTCF binding sites at TAD boundaries did they divide by 2 or how did they do this analysis? Please state this more clearly in the figure legend.

c. for the definition of clusters of CTCF sites, the authors allow for a stretch of DNA and then quantify the number of CTCF sites, which makes sense. But it seems like they use slightly different lengths in different analyses, which is a bit confusing. Can the authors please clearly state the length they used in the figure legend or main text for the analysis in question?

d. "The insulating function of CTCF at TAD boundaries is robust to species-specific loss of

conserved binding events" I feel this title makes a bit too strong of a claim. Don't you need Hi-C data in all of the species to make claims about the ability of CTCF to insulate (i.e. regulate the contact probability of genomic loci)?

5. Effective CTCF binding site occupancy

In this paper the authors describe a key role for CTCF binding site clusters and also for the affinity of the CTCF binding sites. If we take a step back, presumably the key thing is not whether or not there is a CTCF motif, but whether or not a single CTCF protein was bound at the site at the exact moment in time when an extruding cohesin moves towards the motif. In other words, one of the key variables is likely to be the "time-averaged occupancy" of a CTCF site. E.g. a site bound 20% of the time and a site bound 60% of the time would probably both result in a MACS-called ChIP-Seq peak. But the 60% site would - presumably and all other things being equal - result in better TAD/loop stabilisation.

I bring this up, because 2 very recent papers (PMID: 31204999, 31205001) recently estimated the effective occupancy of CTCF sites, albeit in different cell types. I wonder if it would be worth discussing the role of computationally estimated binding affinity and clustering in light of these, as a means for increasing the probability that a CTCF protein would actually be present when a cohesin extrudes.

This is perhaps a bit speculative and the authors should feel free to disregard this suggestion if they do not think it will fit in.

===

This box is for your report. All comments included here will be passed to the authors.

===

Please consider the following questions:

- Are the methods appropriate to the aims of the study, are they well described, and are necessary controls included? If not, please specify what is required.

- Are the conclusions adequately supported by the data shown? If not, please explain

- Are sufficient details provided to allow replication and comparison with related analyses that may have been performed? If not, please specify what is required.

- Does the work represent a significant advance over previously published studies?

- Is the paper of broad interest to others in the field, or of outstanding interest to a broad audience of biologists?

**Reviewer 2**

**Are you able to assess all statistics in the manuscript, including the appropriateness of statistical tests used?**

Yes, and I have assessed the statistics in my report.

**Comments to author:**

Kentepozidou et al. report that evolutionary conservation of CTCF site clusters is critical to the functional stability of higher order chromatin structure.

In the manuscript the authors address the problem that there are many CTCF- site with diverse functions throughout the genome, but only a small fraction of them occur at TAD boundaries. This makes it challenging to delineate the precise role of CTCF binding in stabilizing TAD structures. In this manuscript the authors choose an evolutionary approach and exploit the natural genetic variation across different species to assess how CTCF binding patterns contribute to the establishment of TAD. Using these 'experiments of nature' they show that TAD boundaries are characterized by clusters of both evolutionarily old and young CTCF binding sites and that the majority of clustered CTCF sites at TAD boundaries colocalizes with cohesin.

The experiments are performed well, the dataset is interesting, and the results are conclusive. However, the question of CTCF binding and conservation at TAD boundaries is not entirely new. The manuscript could be of interest to the readers of Genome Biology, but the current version fails to extract much novel biological insights from the dataset and remains descriptive.

I have a few comments on the current ms:

1)	Why did they choose liver as a tissue?

2)	How was TAD boundary calling done for the enrichment of CTCF conservation at boundaries?

3)	Did the authors check the orientation of conserved CTCF site at TAD?

4)	The authors show quantitative differences of ChIP enrichment between CTCF binding at boundaries and in non-boundary regions. Could they validate theses quantitative changes for a selected regions by ChIP qPCR?

5)	The fact that CTCF sites and the cohesin subunit RAD21 cluster together is not surprising and also not new. Which role does the CTCF orientation play?

**Authors Response**

**Point-by-point responses to the reviewers’ comments:**

We thank the reviewers for their careful reading of the manuscript and helpful comments on the work and its presentation. We have addressed all of the points below and added appropriate changes to the manuscript including figures showing the raw data, novel analyses and examples. We have also added novel experiments to strengthen our claims regarding CTCF binding strength.

Reviewer #1: Review of "Clustered CTCF binding is an evolutionary mechanism to maintain topologically associating domains"

The present paper by Kentepozidou et al. looks at genomics data across five different mouse species to study how variation in CTCF binding affects various functional aspects, such as TADs, gene expression and association with cohesin. I believe the paper is topical and of general interest - CTCF has emerged as the main architectural protein and regulator of 3D genome organization in mammals and is currently under intense interest. A general conundrum in the field has been the fact that whereas almost all TADs are demarcated by CTCF binding sites, most CTCF binding sites do not actually form TADs. The identification of a key role for clustered CTCF binding sites by Kentepozidou et al. offers perhaps a partial solution to this conundrum, which I believe makes it likely that this paper will be of significant interest to the field and beyond.

Recently, there have been a number of perturbation experiments performed where CTCF is acutely depleted and/or specific CTCF binding sites are deleted in the genome and then the effect on 3D genome organization, TADs, gene expression, etc. is assessed. These have been really important and informative. Kentepozidou takes a slightly different approach by exploiting the natural variation between 5 different mouse species. Here, evolution has performed a "natural experiment", which allows the authors to assess how specific CTCF binding sites affect function. The authors are not the first to do this - several previous studies have looked at CTCF across species and/or studied the evolution of CTCF binding sites - but I do think that the present study makes a number of significant and original observations, which go well beyond what has previously been shown.

Finally, as a random tangent, I must say I was quite surprised and intrigued by Figure 4A. I think this is quite an interesting observation.

*>> We thank the reviewer for recognising the contributions, value and interest of the observations in this study.*

1. Show me the data please

a. The authors generally do a good job of showing complicated and large datasets and analyses in simple and fairly easy to understand figures. But I think they take it a bit too far. The whole basis of this paper is ChIP-Seq data, but they never actually show us the raw data. I'd like to see a main figure (probably in Figure 1) that shows raw ChIP-Seq tracks (e.g. genome browser) for all 5 mouse species.

I'd also like to set other ChIP-Seq data plots in the supplementary figures additionally.

*>> We have added a panel to Figure 1 (Fig. 1A) showing raw ChIP-seq tracks from the three biological replicates of each of the five species. The figure illustrates an orthologous region among the five mouse species. Therefore, the figure illustrates both reproducibility of peaks and examples of CTCF binding sites that are conserved among the Mus species.*

*Furthermore, we added another figure (Fig. 5), where we show ChIP-seq tracks of the raw data for three examples of TAD boundary regions with clustered CTCF sites. The figures illustrate examples of lineage-specific losses or gains of new sites next to species-conserved sites.*

b. It seems like the authors use the Hi-C data from Vietri Rudan. But this is not Hi-C data for all 5 mouse species and while it is true that TADs are somewhat conserved, some TADs are not. And the fact that the authors do not have high-res Hi-C data for all 5 mouse species is a serious limitation, that I believe that should openly stated in the main text to make this clear to the reader. In fact, I'd like to see the raw Hi-C data that the authors used plotted as contact maps as well to allow the reader to assess the quality.

*>> We apologise if there was any confusion about the source of the Hi-C as this was completely unintended. We have now clearly stated in the text that we used published Hi-C data from one of the five mouse species (C57BL/6J; page 6, lines 10-14). The key results regarding evolutionary clustering of CTCF binding sites at TAD boundaries in all species (Fig. 4A) were also confirmed in C57BL/6J independently (Fig. S9A) where high resolution Hi-C data exists.*

*We have also added a supplementary figure (Fig. S2) showing example regions of the C57BL/6J Hi-C contact maps, which were visualized with Juicebox (Durand et al., 2016).*

2. How do the replicates compare?

Since the foundation of this paper is ChIP-Seq data, the quality and reproducibility of this data is key for substantiating the conclusions, but the authors do not actually show this. They say they performed 3 biological replicates. Please show the following:

a. ChIP-Seq tracks for all 5 species and each replicate individually (probably in Supplement).

*>> This is addressed in comment 1a above by adding Figure 1A, which shows raw ChIP-seq tracks from the three biological replicates of each of the five species.*

b. Please perform reproducibility analysis between replicates

c. Please show the binding heatmaps (rows = different CTCF sites, columns = +/- e.g. 2kb) for each replicate individually and also Venn Diagrams between replicates

*>> We have included Venn diagrams and binding heatmaps showing the high level of biological reproducibility of peaks among replicates (Fig. S1).*

d. Please include a supplementary figure or table with summary data for all the replicates: number of reads per replicate, number of identified peaks per replicate (e.g. MACS) and other useful summary data

I understand that the raw data is available for download, but I still believe it is essential that the authors perform these analyses and shown them in an easy-to-assess manner in their manuscript.

*>> We thank the reviewer for this suggestion. We have calculated the mapping and peak calling statistics and included them in Supplementary Table S1: Library quality statistics.*

3. Language

The paper is generally well-written and easy to follow. It is also not too long. But there were a few places where I found the language confusing, including:

Abstract: "Our analyses reveal that CTCF binding is maintained at TAD boundaries by an equilibrium of selective constraints and dynamic evolutionary processes." - can evolution really be said to be in an equilibrium? This implies a thermodynamic stability. I would suggest re-writing or explaining what is meant by an evolutionary equilibrium.

*>> We agree that “equilibrium” could be misunderstood. We therefore replaced “equilibrium” with “balance” (page 2, line 14). We hope this now better reflects that on the one hand we observe selective constraints at the genomic regions of TAD boundaries, such as potentially negative selection of LINE insertions, conserved CTCF binding sites, and conserved clusters of CTCF sites. On the other hand, we also observe rather dynamic evolutionary processes which allow for some extent of plasticity of the CTCF binding profile at TAD boundaries. These dynamic processes are the gains and losses of particular CTCF sites at TAD boundaries, which sometimes form clusters.*

"Dynamic conservation of CTCF site clusters is an apparently important feature …" What does "Dynamic Conservation" mean? Dynamic implies change and conservation implies the opposite of change? Please explain.

Page 19, line 6: "dynamically conserved regions": again it's not very clear to me what "dynamic conservation" means. See also above.

*>> We have added examples of this dynamic conservation in Figure 5. If a genomic region in one species harbours a cluster of CTCF sites, its orthologous region in the other species is very likely to also harbour a cluster of CTCF sites. Furthermore, most individual binding sites in the cluster are usually conserved across the species. That is why we call the clusters “conserved”. However, such a conserved cluster might also have divergent sites in some of the species, as a result of lineage-specific gains or losses of particular sites. As a result, a cluster might be found at an orthologous region among the species, yet it can contain a slightly different number and position of CTCF sites in each species. This is why we call them “dynamically conserved”. We have now also elaborated on this in the manuscript text (page22, lines 8-11).*

4. Miscellaneous Comments

a. binding affinity. I slightly object to the use of the term "binding affinity" in a purely computational study. I understand that there are ways of algorithmically guestimating these, and I like the fact that the authors did this analysis, but I still think the authors should clearly caveat this and try to avoid using the term "binding affinity" unless they have actually measured it. At least say "computationally estimated binding affinity" or something along those lines.

*>> As a point of note, although the first and senior authors are from a computational institute, this study includes extensive new experimental data and half of the paper's authors are experimental biologists. This said, we agree with the point that we did not conduct experiments to specifically measure binding affinity and we have updated the text to make it clearer that this result refers to predicted binding affinity (page 7, lines 7-8, 11).*

*We also noted in this section that the computational predictions of motif binding affinity agree with the observed experimental ChIP-seq signal enrichment at the various CTCF binding sites. In addition, we have now included a comparison of mapped read counts between TAD boundary-associated and non TAD boundary-associated CTCF peaks (Fig. S6), showing a higher coverage of the former ones. Taken together, these independently acquired results support and reinforce each other.*

b. Figure 4D. I was confused about how this was defined. TADs are adjacent and in the simplest model demarcated by convergent CTCF sites. In the simplest model then we would expect there to be at least 2 CTCF sites at the boundary between two TADs; one for the left TAD boundary and one for the right TAD boundary. As such, we should perhaps divide by 2 if we want to quantify the number of CTCF sites per TAD at a TAD boundary.

When the authors report the number of CTCF binding sites at TAD boundaries did they divide by 2 or how did they do this analysis? Please state this more clearly in the figure legend.

*>> We have clarified this in the corresponding figure legend. In general, we define one TAD boundary region between two adjacent TADs as the first nucleotide of the downstream TAD +/- a 50kb window. We do not define two different boundaries for the upstream and downstream TAD respectively. Therefore, we do not divide the number of CTCF sites by two.*

c. for the definition of clusters of CTCF sites, the authors allow for a stretch of DNA and then quantify the number of CTCF sites, which makes sense. But it seems like they use slightly different lengths in different analyses, which is a bit confusing. Can the authors please clearly state the length they used in the figure legend or main text for the analysis in question?

*>> Thank you for the remark and we apologize if the distinction between TAD boundary and TAD boundary regions was not clear enough. We have revised the legend of Fig. 1D, and all instances of TAD boundary and TAD boundary regions in the manuscript to make the distinction as clear as possible.*

*Specifically, we use two different approaches when referring to association of CTCF sites to a TAD boundary, depending on the question being asked. These are the following:*

*a) When referring to the distance of a CTCF site from a TAD boundary, we define the TAD boundary between two adjacent TADs as the first nucleotide of the downstream TAD. Then, we count the distance of the CTCF site from this one-nucleotide-long TAD boundary. This is now consistently referred to as “TAD boundary” in the manuscript.*

*b) When looking at CTCF sites that are potentially associated with the demarcation and/or maintenance of a TAD boundary (TAD boundary-associated sites), we use a window of +/- 50kb around this single-nucleotide long TAD boundary (as defined above) between two adjacent TADs. We define this window as the “TAD boundary region” and see how many binding sites this region harbours.*

*Regarding this approach, we hope the following additional background information further clarifies our reasoning.*

*It is generally challenging to define the exact genomic coordinates of a TAD boundary due to the resolution of Hi-C data and the complexity of genome biology. Some relevant limitations to consider are:*

*a. Unlike genes, TADs are not precisely delimited functional units/elements with absolutely fixated start and end coordinates. They are rather large regions within which there is a high frequency of genomic interactions. These interactions are quite dynamic and contribute to gene regulation.*

*b. TADs are dynamic structures themselves, given that they can disappear and then re-form along the cell cycle (Oomen et al., 2019).*

*c. The contact maps we use to study the chromatin structure are snapshots of the genomic interactions at a specific temporal point and of a population of potentially variable cells (in terms of contact interactions). This means that the TAD boundaries that we observe are also a consensus within a population of cells.*

*d. The Hi-C data we use are of a limited resolution. The TADs called by Vietri-Rudan were based on generated interaction matrices of resolutions 12,500, 25,000, 50,000, 100,000, 250,000, 500,000, and 1,000,000 bp. This means that when referring to a “one-nucleotide-long TAD boundary”, the actual boundary would be within a window of at least +/-12.5 kb around this nucleotide. In addition, even this bin might not be accurate if the sequencing depth of the Hi-C restriction fragments is not adequately large to allow for a highly accurate estimate of the interaction frequency between these fragments.*

*In conclusion, given the available data, we can only roughly define the region where a TAD boundary is located. For this reason, and pointing back to comment 4b, we can identify CTCF sites that occur in the region of a TAD boundary, but we cannot always be sure about whether a site belongs to the upstream or the downstream TAD. One thing that is helpful in many cases is to look at the orientations of the CTCF binding sites. However, even this is not always trivial, as many CTCF sites contain more than one motif that can have different orientations, some CTCF sites can have multiple functions, while the clustering of neighbouring CTCF sites makes this task of assigning CTCF sites to adjacent TADs more complicated.*

d. "The insulating function of CTCF at TAD boundaries is robust to species-specific loss of

conserved binding events" I feel this title makes a bit too strong of a claim. Don't you need Hi-C data in all of the species to make claims about the ability of CTCF to insulate (i.e. regulate the contact probability of genomic loci)?

*>> We have now softened this claim with relevant corrections in the text (page 18, lines 21-22; page 21, line 8) and moved a more speculative comment about insulating function to the discussion.*

5. Effective CTCF binding site occupancy

In this paper the authors describe a key role for CTCF binding site clusters and also for the affinity of the CTCF binding sites. If we take a step back, presumably the key thing is not whether or not there is a CTCF motif, but whether or not a single CTCF protein was bound at the site at the exact moment in time when an extruding cohesin moves towards the motif. In other words, one of the key variables is likely to be the "time-averaged occupancy" of a CTCF site. E.g. a site bound 20% of the time and a site bound 60% of the time would probably both result in a MACS-called ChIP-Seq peak. But the 60% site would - presumably and all other things being equal - result in better TAD/loop stabilisation.

I bring this up, because 2 very recent papers (PMID: 31204999, 31205001) recently estimated the effective occupancy of CTCF sites, albeit in different cell types. I wonder if it would be worth discussing the role of computationally estimated binding affinity and clustering in light of these, as a means for increasing the probability that a CTCF protein would actually be present when a cohesin extrudes.

This is perhaps a bit speculative and the authors should feel free to disregard this suggestion if they do not think it will fit in.

*>> We thank the reviewer for pointing out these results. We agree that they are relevant to our work and have mentioned them appropriately in the context of the discussion (page 22; lines 21-33).*

Reviewer #2: Kentepozidou et al. report that evolutionary conservation of CTCF site clusters is critical to the functional stability of higher order chromatin structure.

In the manuscript the authors address the problem that there are many CTCF- site with diverse functions throughout the genome, but only a small fraction of them occur at TAD boundaries. This makes it challenging to delineate the precise role of CTCF binding in stabilizing TAD structures. In this manuscript the authors choose an evolutionary approach and exploit the natural genetic variation across different species to assess how CTCF binding patterns contribute to the establishment of TAD. Using these 'experiments of nature' they show that TAD boundaries are characterized by clusters of both evolutionarily old and young CTCF binding sites and that the majority of clustered CTCF sites at TAD boundaries colocalizes with cohesin.

The experiments are performed well, the dataset is interesting, and the results are conclusive. However, the question of CTCF binding and conservation at TAD boundaries is not entirely new. The manuscript could be of interest to the readers of Genome Biology, but the current version fails to extract much novel biological insights from the dataset and remains descriptive.

*>> We thank the reviewer for their comments and suggestions for improvement of our results and their presentation.*

I have a few comments on the current ms:

1) Why did they choose liver as a tissue?

*>> We and other authors have used liver tissue for ChIP-seq and Hi-C experiments because liver is a relatively homogenous primary tissue (approximately 80-85% hepatocytes) and thus an ideal choice for experimental assays that require tens to hundreds of thousands of cells. Our results build on previously published work from our group and others including Schmidt et al., 2012; Thybert et al., 2018; and Vietri Rudan et al., 2015.*

2) How was TAD boundary calling done for the enrichment of CTCF conservation at boundaries?

*>> A TAD boundary between two adjacent TADs was defined as the first nucleotide of the adjacent TAD. Wherever we report the distance of a CTCF site from a TAD boundary (e.g in Fig. 1D, or 4A), we refer to the distance of the site to this single nucleotide. However, in cases where we report for example how many CTCF sites are harboured in a TAD boundary region (e.g. Fig. 4D, 4E), we have extended a window of +/-50kb around this nucleotide. We have now elaborated on this in the legend of Fig. 1D.*

3) Did the authors check the orientation of conserved CTCF site at TAD?

*>> We performed orientation analyses and found that 92.3% of the Mus-conserved CTCF peaks contain motifs with conserved orientation in all five species. We have now included this piece of information in the manuscript (page 5, lines 8-9). For the Mus-conserved sites specifically at TAD boundaries, the percentage of conserved peaks with species-conserved motif orientations is very similar (93.1%). We thank the reviewer for this question, which we believe has strengthened our claims of overall CTCF conservation.*

4) The authors show quantitative differences of ChIP enrichment between CTCF binding at boundaries and in non-boundary regions. Could they validate theses quantitative changes for a selected regions by ChIP qPCR?

*>> We thank the reviewer for the suggestion for validation of quantitative changes. The samples we originally used for the ChIP-seq experiments were used up completely in the initial experiments, which excluded the option of performing additional qPCR validation.*

*However, to provide further evidence of quantitative differences of ChIP enrichment at boundary versus non boundary regions, we performed ChIP-seq experiments in two additional biological replicates of C57BL/6J, calculated the mapped read counts for peaks of each category and showed that the boundary-associated ones generally have higher stacks of mapped reads (Fig. S6). To increase the robustness of our observation we performed this analysis of mapped read counts in the original samples, and the two additional independent CTCF ChIP-seq libraries. Our observations were consistent among all five biological replicates.*

5) The fact that CTCF sites and the cohesin subunit RAD21 cluster together is not surprising and also not new. Which role does the CTCF orientation play?

*>> We did not intend to claim that we discovered a novel association between CTCF and cohesin and we apologise if this was implied in the manuscript. We included this analysis in support of the observation that clustered CTCF sites have stronger signatures of functionality than singleton CTCF sites, given what is already known about CTCF biology. We have updated the text to make this clearer.*

*Concerning the orientations of clustered CTCF sites, we further investigated the orientations of species-specific CTCF site gains in clusters. We found that 70% of the newly acquired species-specific CTCF sites have the same orientation as the consensus orientation of the Mus-conserved (5-way) sites included in the same cluster. These newly acquired species-specific sites potentially have an additive effect in binding and stabilizing CTCF in the region or could be used interchangeably with their neighbouring CTCF sites to bind CTCF molecules that unbind and reload on the genome during molecular processes like gene transcription. We have now added further information in the manuscript (page 14, lines 8-17) referring to these observations of ours.*

*REFERENCES*

*Durand, N.C., Robinson, J.T., Shamim, M.S., Machol, I., Mesirov, J.P., Lander, E.S., and Aiden, E.L. (2016). Juicebox Provides a Visualization System for Hi-C Contact Maps with Unlimited Zoom. Cell Syst.*

*Oomen, M.E., Hansen, A.S., Liu, Y., Darzacq, X., and Dekker, J. (2019). CTCF sites display cell cycle-dependent dynamics in factor binding and nucleosome positioning. Genome Res.*

*Schmidt, D., Schwalie, P.C., Wilson, M.D., Ballester, B., Gonçalves, A., Kutter, C., Brown, G.D., Marshall, A., Flicek, P., and Odom, D.T. (2012). Waves of retrotransposon expansion remodel genome organization and CTCF binding in multiple mammalian lineages. Cell 148, 335–348.*

*Thybert, D., Roller, M., Navarro, F.C.P., Fiddes, I., Streeter, I., Feig, C., Martin-Galvez, D., Kolmogorov, M., Janoušek, V., Akanni, W., et al. (2018). Repeat associated mechanisms of genome evolution and function revealed by the Mus caroli and Mus pahari genomes. Genome Res. 28, 448–459.*

*Vietri Rudan, M., Barrington, C., Henderson, S., Ernst, C., Odom, D.T., Tanay, A., and Hadjur, S. (2015). Comparative Hi-C Reveals that CTCF Underlies Evolution of Chromosomal Domain Architecture. Cell Rep. 10, 1297–1309.*

**Second round of review**

**Reviewer 2**

The authors answered all my questions and included some new analysis. I have no further concerns or questions.
